# Traumatic and non-traumatic bone marrow edema in ankle MRI: a pictorial essay

**DOI:** 10.1186/s13244-020-00900-8

**Published:** 2020-08-17

**Authors:** Pawel Szaro, Mats Geijer, Nektarios Solidakis

**Affiliations:** 1 Department of Musculoskeletal Radiology, Sahlgrenska University Hospital, Gothenburg, Sweden; 2 Department of Radiology, Institute of Clinical Sciences, Sahlgrenska Academy, University of Gothenburg, Gothenburg, Sweden; 3 Department of Clinical Sciences, Lund University, Lund, Sweden

**Keywords:** Bone marrow edema, Ankle trauma, Sports injury, Ankle sprain, Magnetic resonance imaging

## Abstract

Bone marrow edema (BME) is one of the most common findings on magnetic resonance imaging (MRI) after an ankle injury but can be present even without a history of trauma. This article will provide a systematic overview of the most common disorders in the ankle and foot associated with BME.

The presence of BME is an unspecific but sensitive sign of primary pathology and may act as a guide to correct and systematic interpretation of the MR examination. The distribution of BME allows for a determination of the trauma mechanism and a correct assessment of soft tissue injury. The BME pattern following an inversion injury involves the lateral malleolus, the medial part of the talar body, and the medial part of the distal tibia. In other cases, a consideration of the distribution of BME may indicate the mechanism of injury or impingement. Bone in direct contact with a tendon may lead to alterations in the bone marrow signal where BME may indicate tendinopathy or dynamic tendon dysfunction. Changed mechanical forces between bones in coalition may lead to BME. Degenerative changes or minor cartilage damage may lead to subchondral BME. Early avascular necrosis, inflammation, or stress fracture may lead to more diffuse BME; therefore, a detailed medical history is crucial for correct diagnosis.

A systematic analysis of BME on MRI can help to determine the trauma mechanism and thus assess soft tissue injuries and help to differentiate between different etiologies of nontraumatic BME.

## Key points

- BME is a common finding; therefore, a systematic analysis is crucial.
- The knowledge of trauma mechanism allows for more accurate soft tissue diagnosis.
- Bone marrow edema not related to trauma requires a systematic assessment.
- Accurate non-traumatic bone marrow edema analysis enables precise radiological reporting.
- Subchondral bone marrow edema is a sensitive indicator of cartilage lesions.

## Introduction and terminology

### What is bone marrow edema?

**Fig. 1:**
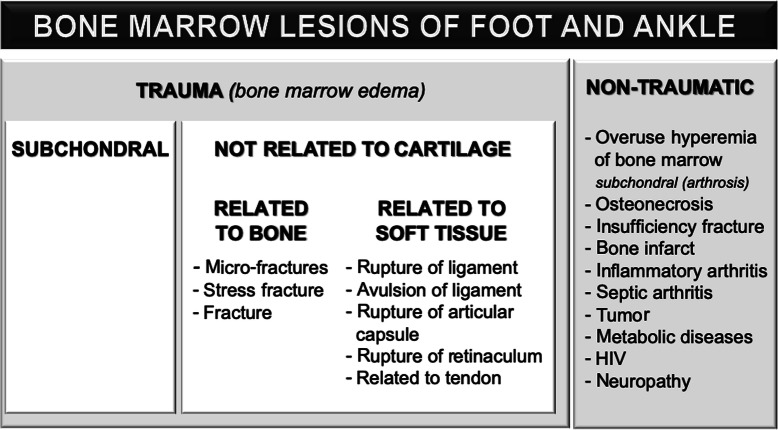
Common etiologies of bone marrow edema in the ankle [1–3]

Magnetic resonance imaging (MRI) after ankle trauma often shows alteration of bone marrow signal with a low signal on T1-weighted and a high signal on T2-weighted and fluid-sensitive sequences (like short tau inversion-recovery (STIR) or fat-suppressed (FS) sequences). This is known as bone marrow edema (BME) or bone bruise (Fig. 1) [1, 2, 4, 5].

BME is associated with capillary leakage and interstitial extracellular fluid accumulation within the bone marrow, which is responsible for the “bruising” of the bone marrow [6]. Patients without injury often show changes with similar morphology on MRI, also called BME. In hyperemic BME, the extracellular fluid comes from the inflamed walls of capillaries [7]. Some authors instead use the term *bone marrow lesion* (BML) in those cases, which is a more universal term and use the term BME for trauma-related cases [8].

The presence of BME is an unspecific but sensitive sign of underlying pathology, so correct and systematic interpretation is crucial. Interpretation of BME is multilevel and usually starts with the patient’s history (traumatic or non-traumatic, acute, or insidious). As mentioned above, the differential diagnosis of BME is very wide (Fig. 1) [2].

## Imaging protocol

An MR protocol for ankle imaging should include sequences in all three orthogonal planes and an axial oblique plane if the flexor tendons need more detailed analysis. FS imaging with T2- or proton-density (PD) weighted images is mandatory, as is the inclusion of at least one T1-weighted sequence for bone marrow evaluation. The exact choice of scan planes and weighting depends on the suspected pathology and on local traditions and preferences.

## Bone marrow edema after overuse, minor or major trauma

**Fig. 2:**
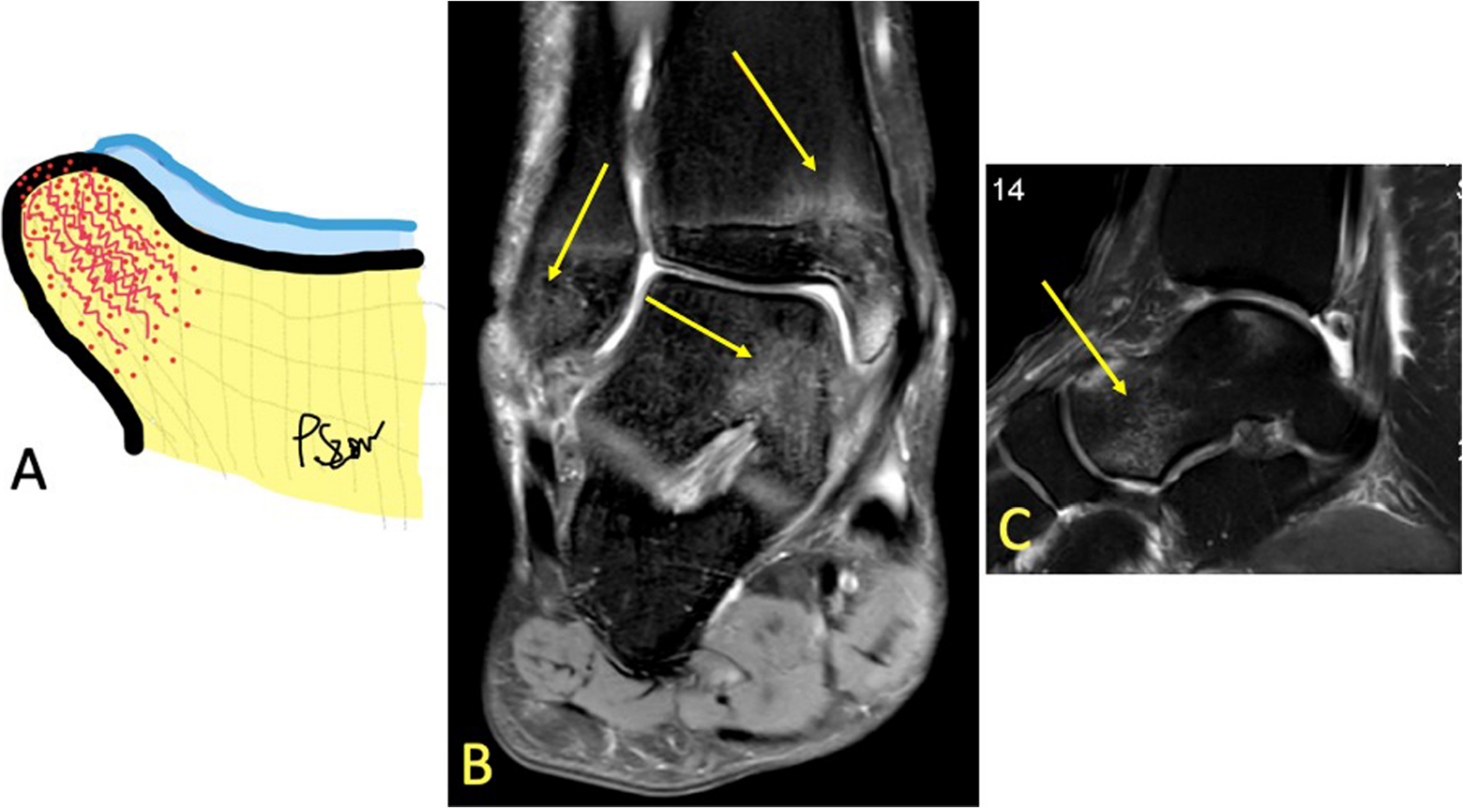
A 20-year-old soccer player presented after an ankle sprain during a soccer match with suspicion of a fracture in the lateral malleolus. MRI showed bone bruise in the tibia, fibula, and talus (arrows). It revealed no fracture of the lateral malleolus

Overuse injuries to the bones and soft tissue structures arise during repeated minor injuries, which are, however, too weak to cause a rupture of tendon or a full fracture. Overload injuries are especially common in sports such as running. However, the clinical manifestation of these lesions may be nonspecific. Overuse lesions occur in relatively constant locations like the subchondral part of talar trochlea. Stress reaction can often be seen in the distal tibia, distal fibula, or the calcaneus. The anterior impingement syndrome may be caused by overuse and micro-injuries. Overuse of the insertion of the Achilles tendon or plantar fascia may appear as BME in the calcaneus [3]. BME associated with overuse or trauma (Fig. 2) usually resolves after a few months (usually 3 months); however, the clinical symptoms disappear sooner, usually after 6 weeks [6, 8, 9]. Sometimes it may take longer for the BME to resolve [1].

Costa—Paz et al. [10] classify BME into three types:

- Type I: Diffuse or reticular BME, at some distance from the articular cartilage
- Type II: Localized or geographic BME, often with a convex margin and contiguous to the articular cartilage or bony outline
- Type III: The BME often has slight deformation or disruption of the bony outline

This classification helps to determine the cause of the BME: type I usually corresponds to an injury from a contrecoup mechanism making it more extensive, whereas type II usually indicates trauma to the ligament attachment, articular capsule, or retinaculum and therefore is more localized. Type III is often associated with a fracture or osteochondral lesion; thus, it may have a different extent [5, 11]. The distribution of BME seen in specific types of injury thus represents one of the most useful differential diagnostic clues in ankle trauma.

**Fig. 3:**
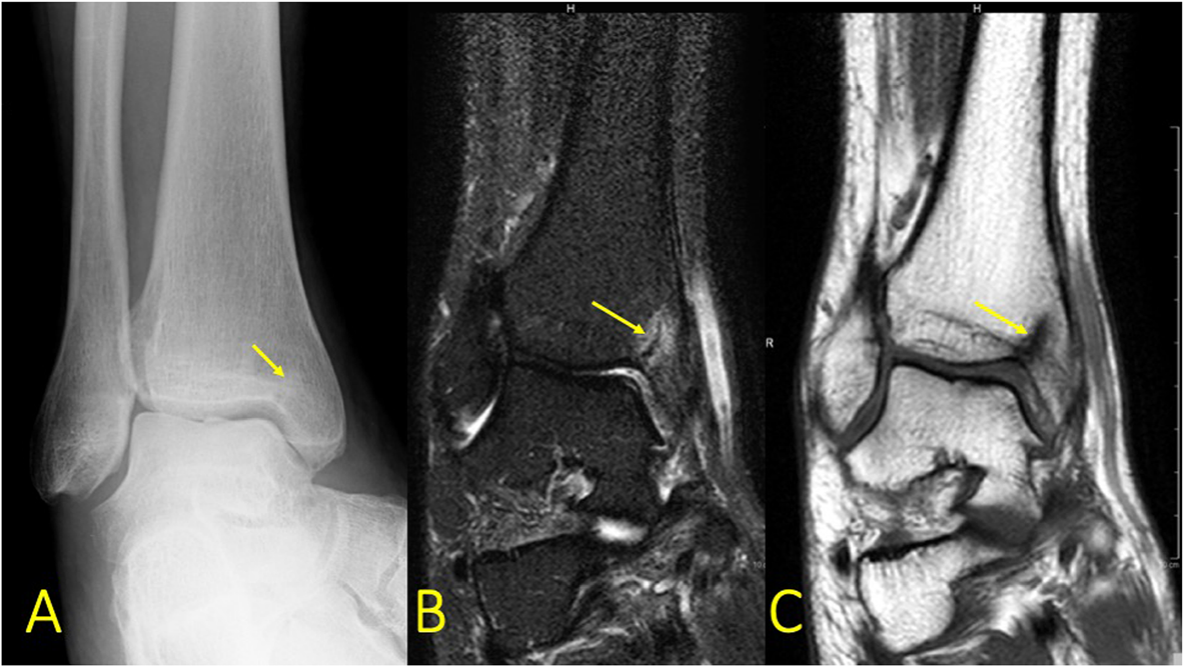
A 57-year-old male with pain in the medial malleolus without a history of trauma. **a** Radiograph, mortise view, **b** PD FS coronal image, and **c** T1-weighted coronal image. MRI showed an occult fracture of the medial malleolus (arrow) which in retrospect was visible on the radiographs. There is a minor irregularity of the cartilage signal in the subchondral central part of the talar trochlea, probably after previous injuries

By firstly determining if there is BME on only one side of the ankle joint or it is multifocal and secondly the type of BME, the BME pattern can reveal the mechanism of injury [1, 9]. The absence of a hypointense line on T1-weighted images excludes a complete fracture which needs a different treatment (Fig. 3).

**Fig. 4:**
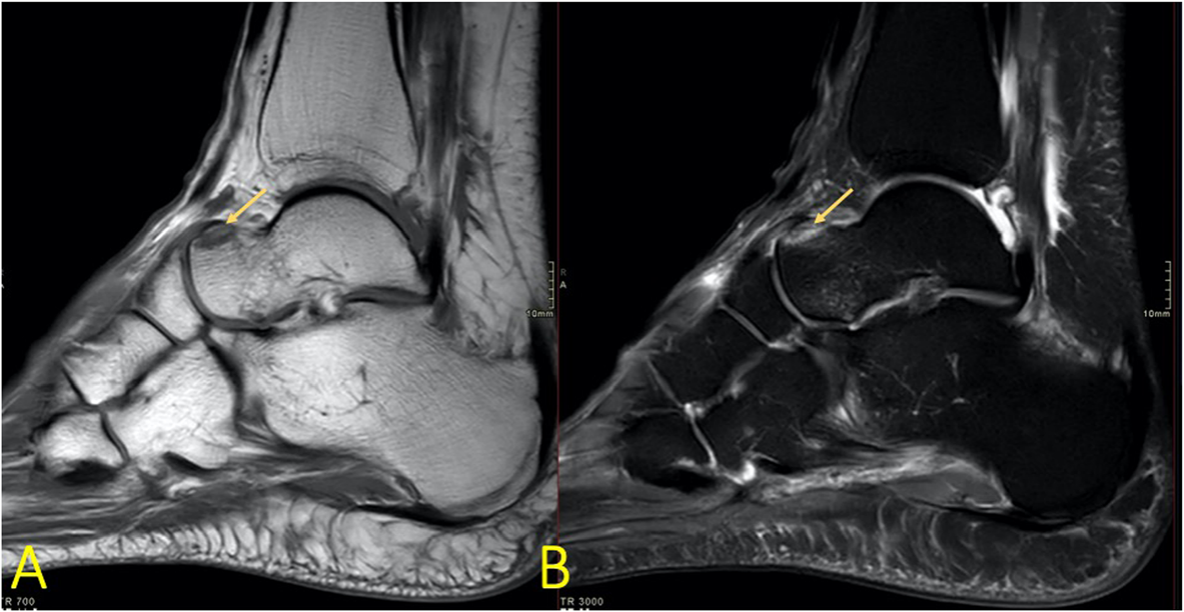
A 19-year-old soccer player sustained an ankle sprain during a soccer match with suspicion of rupture of the anterior tibiofibular ligament. MRI showed avulsion (arrow) of the talar attachment of the dorsal talonavicular ligament

**Fig. 5:**
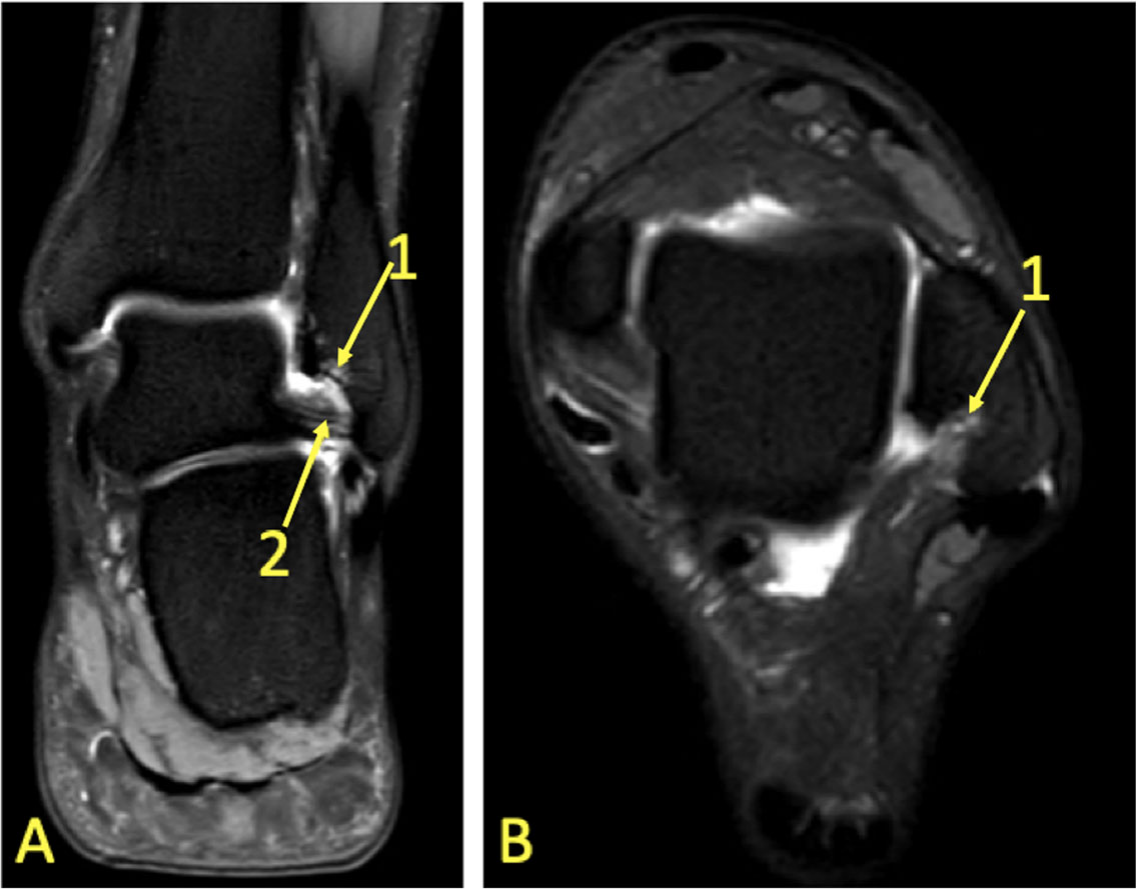
A 42-year-old male with 9-month history of ankle pain and suspicion of an osteochondral lesion. MRI (**a**, **b**) revealed traction cysts at the insertion of the posterior talofibular ligament

The distraction of bone, which is visible in an avulsion injury, causes a linear trabecular disruption in a limited area (Fig. 4). Sometimes it is visible in chronic overuse of ligaments. When it happens chronically in this area, a “traction cyst” (typically at the insertion of the posterior talofibular ligament) may develop (Figs. 5 and 9).

The localization of the BME may point to the injured soft tissue structure. Early visualization of a low-grade soft tissue injury is challenging on MRI, which is why the BME makes it easier to evaluate the MR examination.

### Ligament and articular capsule rupture

**Fig. 6:**
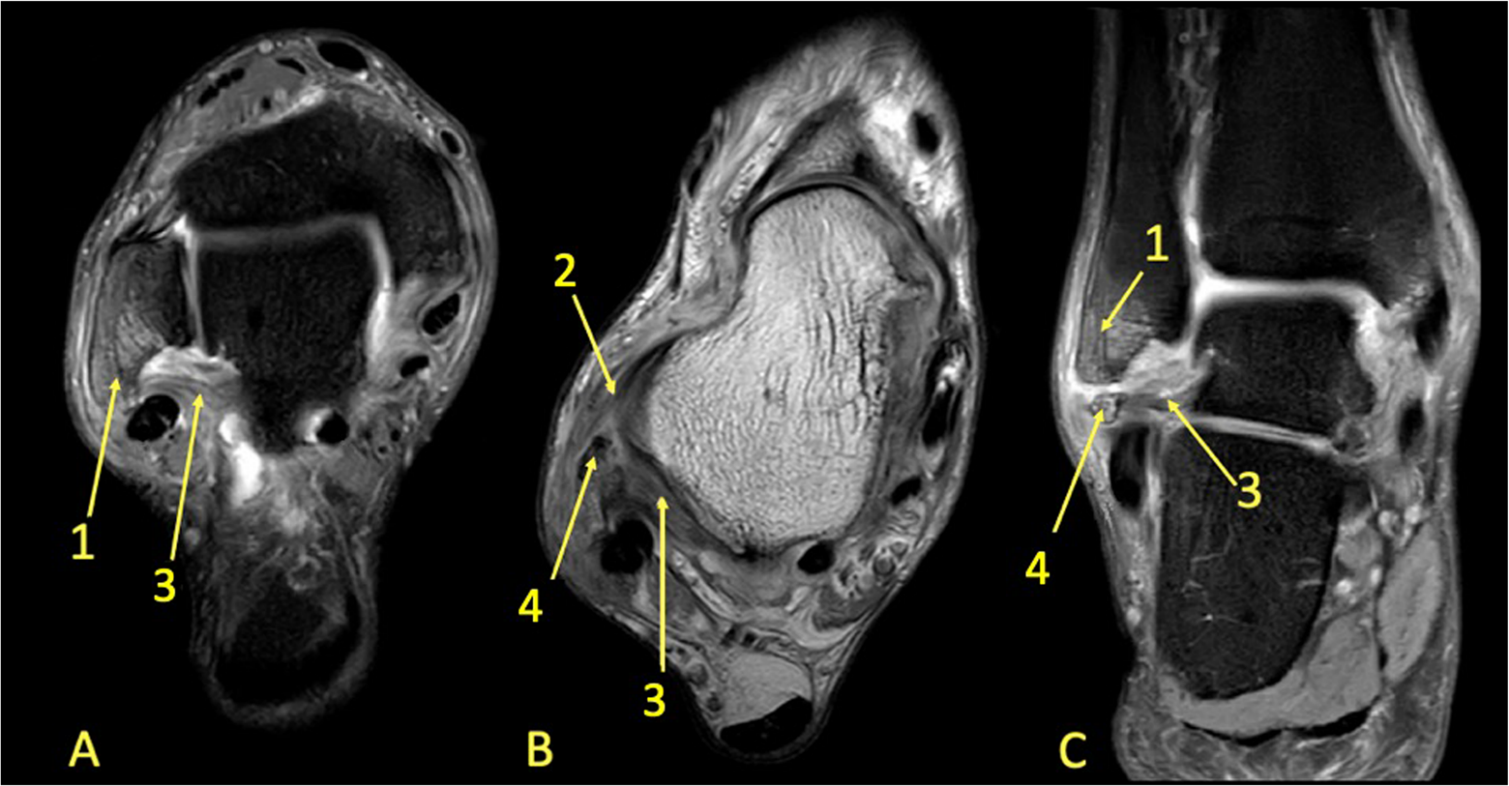
A 24-year-old male imaged on suspicion of an anterior tibiofibular ligament rupture 2 weeks after an ankle injury. MRI showed an avulsion of the anterior talofibular ligament. (1) BME in the lateral malleolus, (2) the anterior talofibular ligament, (3) the posterior talofibular ligament, and (4) an avulsed bone fragment from the lateral malleolus

**Fig. 7:**
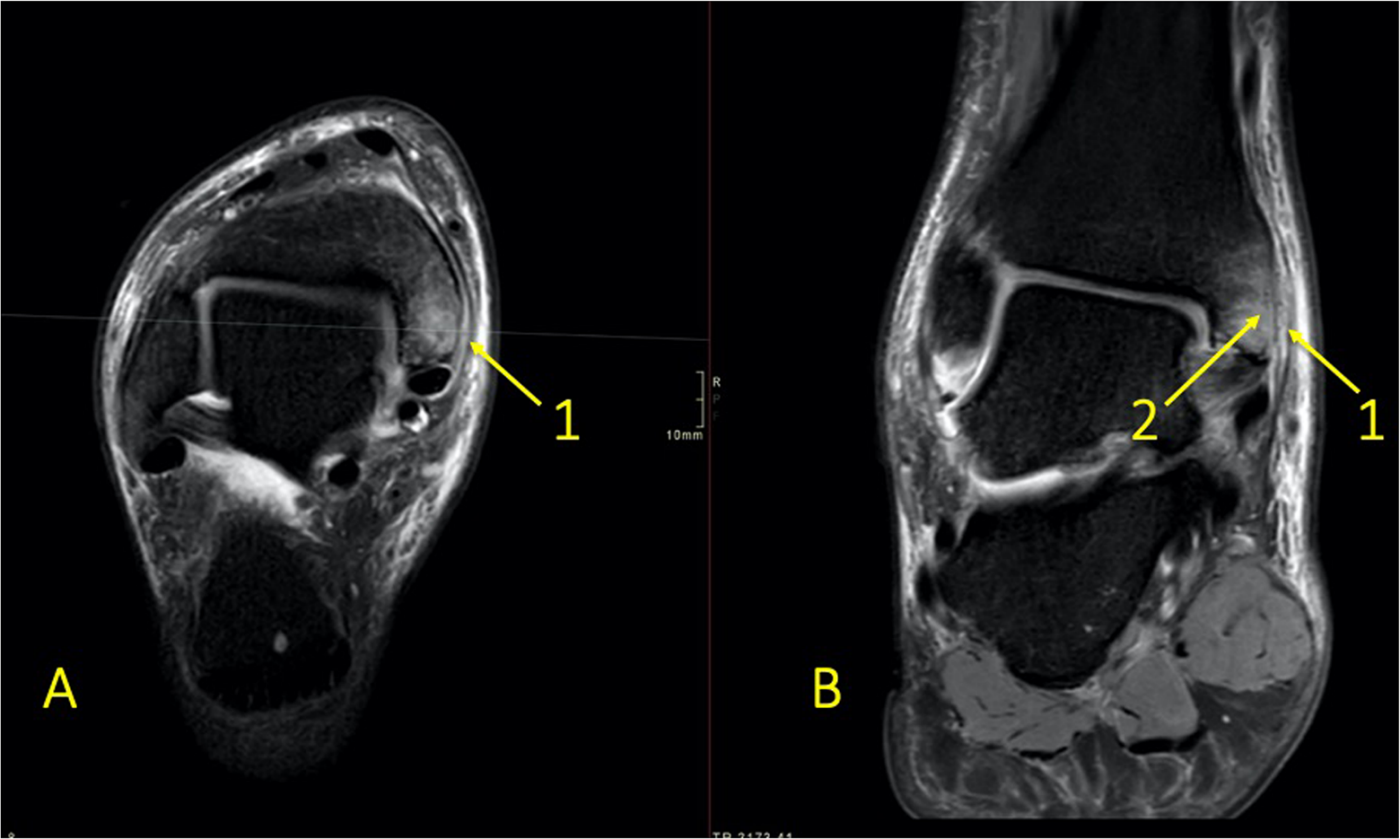
A 39-year-old male imaged 10 days after an ankle injury on suspicion of deltoid ligament rupture. MRI showed (1) partial injury of the flexor retinaculum with (2) BME in the medial malleolus

The distribution of BME is like a footprint of trauma which allows for determination of the trauma mechanism by giving clues to the concomitant soft tissue injuries (Figs. 6 and 7). With an inversion injury (Figs. 2 and 6), an extensive reticular BME is visible in the medial part of the ankle joint, mainly in the medial and postero-medial part of the talus and medial malleolus, because of a contrecoup injury. Usually, small areas of BME are visible on the avulsion side in the lateral malleolus. An eversion injury usually causes an inverse BME distribution. A geographic BME in the medial part of the talar trochlea may indicate an avulsion of the deltoid ligament [8, 9].

### Tendinopathy and enthesopathy

**Fig. 8:**
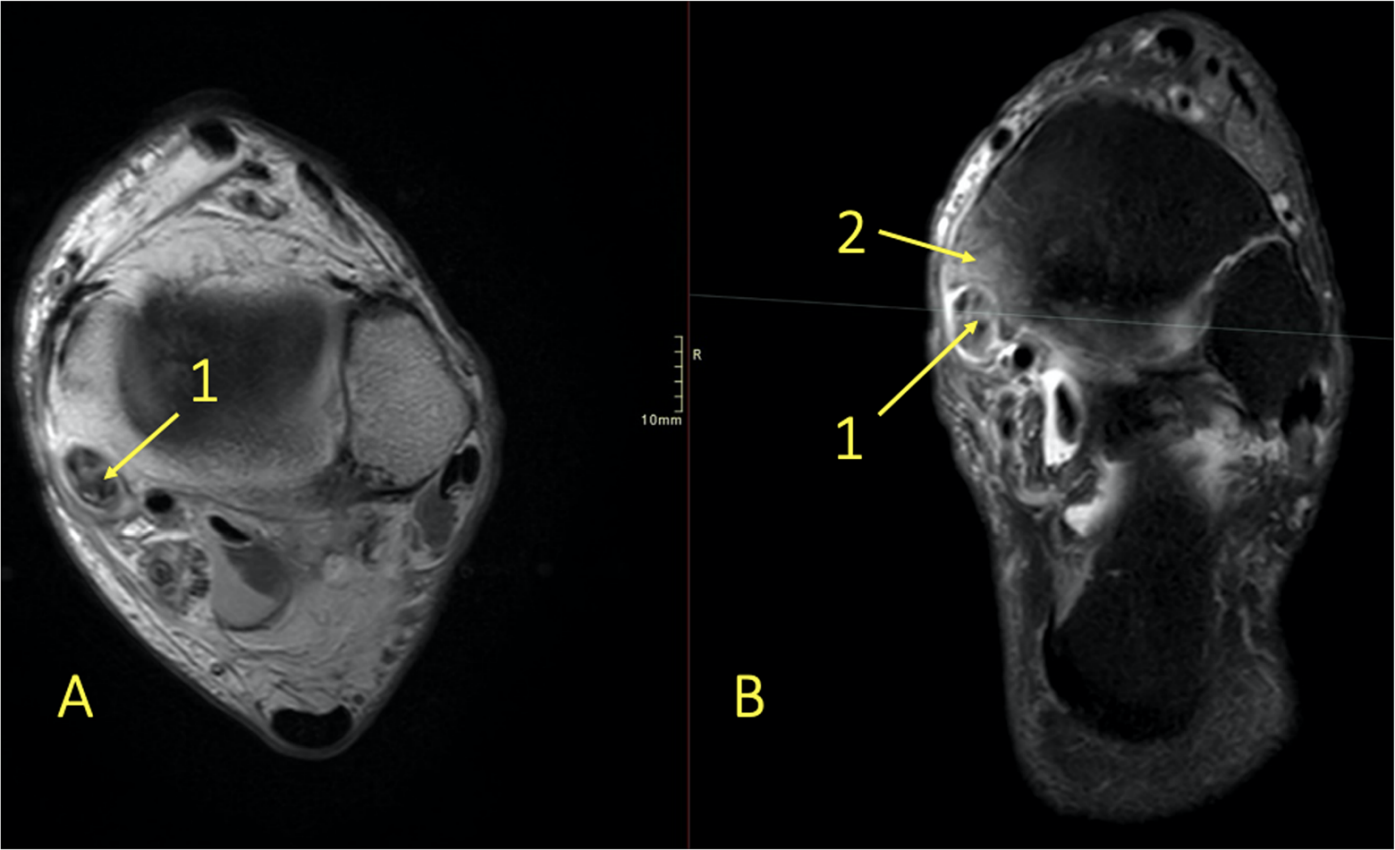
A 65-year-old male with chronic ankle pain after ankle trauma about 3 weeks earlier and a suspected rupture of the tibialis posterior tendon. MRI showed tendinopathy. (1) A partial rupture of the tibialis posterior tendon and (2) BME in the medial malleolus were visible

**Fig. 9:**
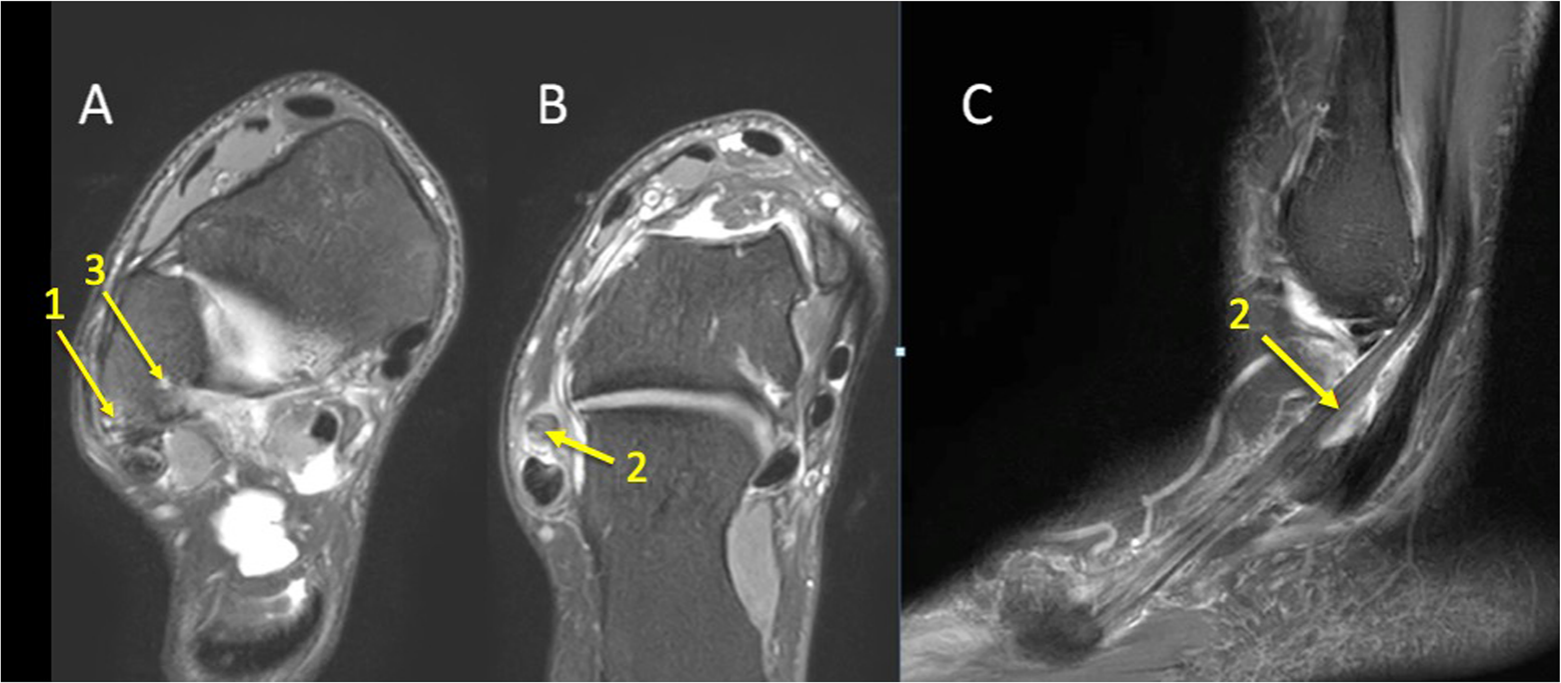
A 59-year-old female with 10-month history of lateral ankle pain and suspicion of an osteochondral lesion. MRI showed tendinopathy of the peroneus brevis tendon. (1) BME in the fibula adjacent to (2) the tendinopathy of the peroneus brevis tendon. (3) A traction cyst at the attachment of the posterior talofibular ligament

**Fig. 10:**
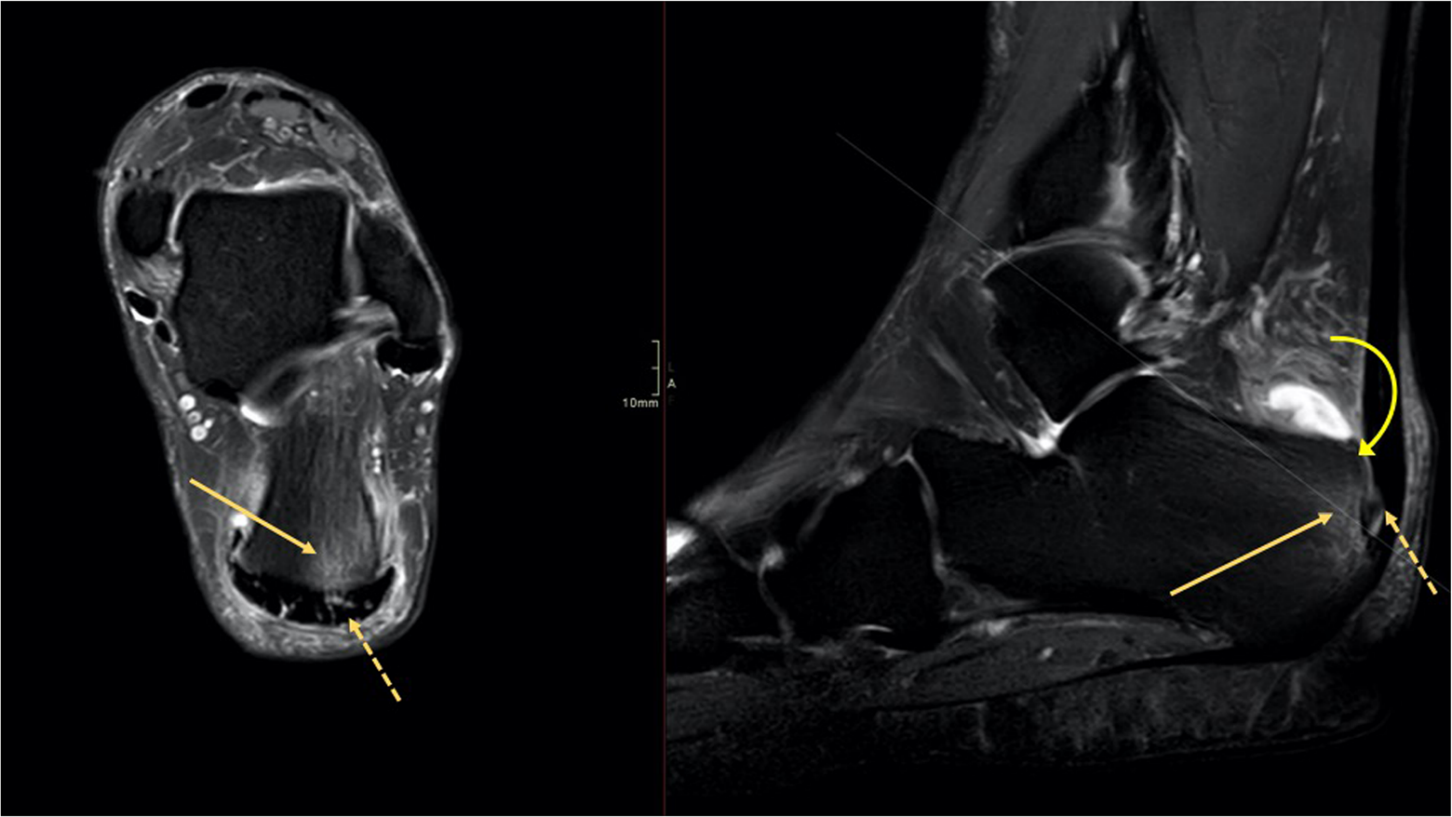
A 49-year-old male with chronic pain at the Achilles tendon enthesis and suspicion of a Haglund deformity. MRI showed enthesopathy of the Achilles tendon (dashed arrow) and BME in the tuber calcanei (arrow) without evidence of a Haglund deformity (curved arrow)

**Fig. 11:**
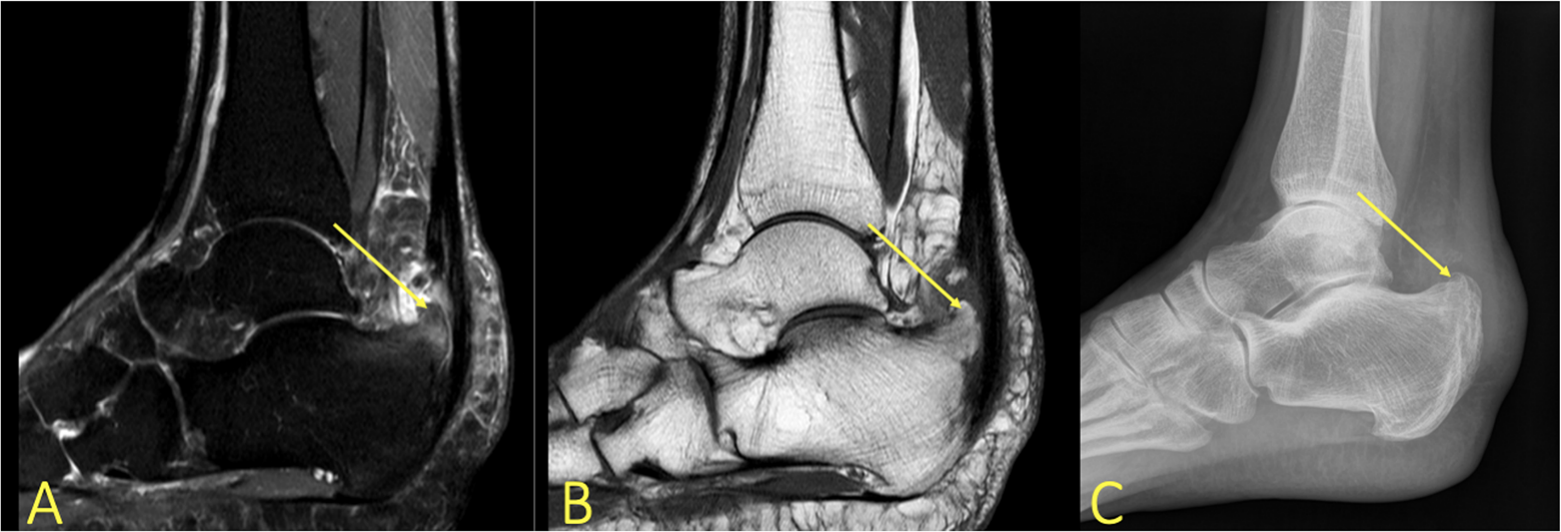
A 62-year-old female with recurring symptoms after previous surgery for a Haglund deformity. MRI and radiography showed a Haglund deformity (arrow) with bone marrow edema again visible on MRI

**Fig. 12:**
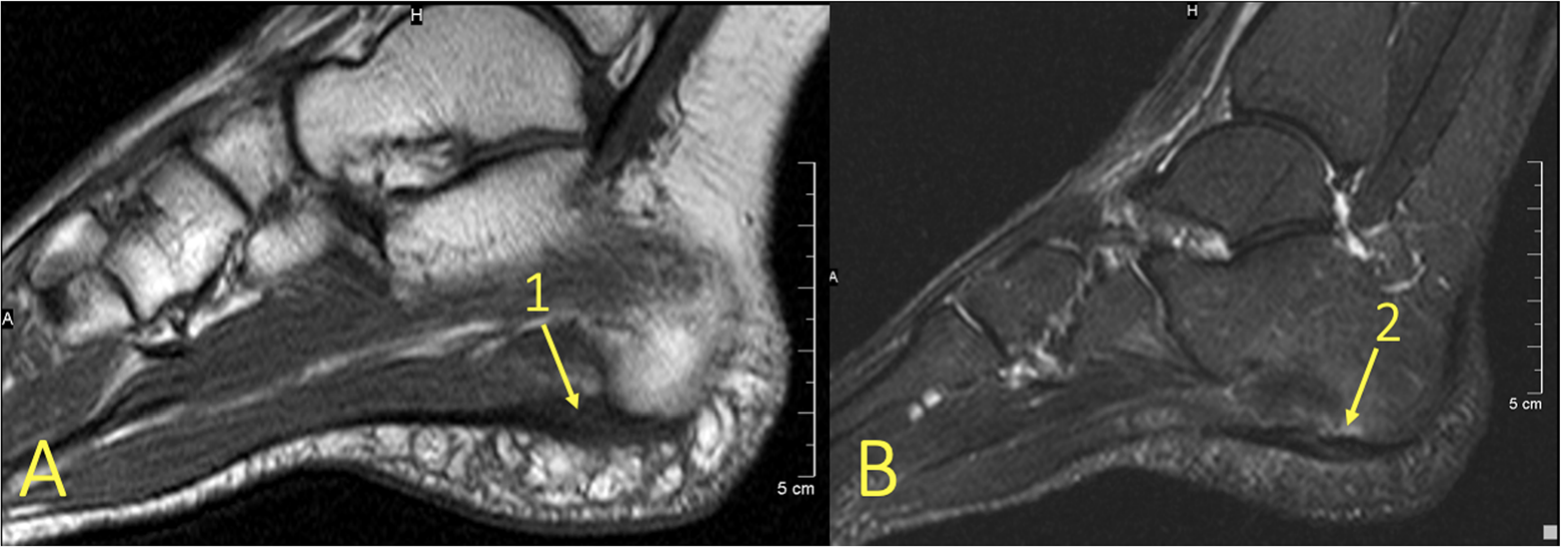
A 43-year-old female with chronic pain in the plantar part of the heel and suspicion of a heel spur and plantar aponeurosis rupture. MRI revealed (1) enthesopathy of the plantar fascia with (2) bone marrow edema in the calcaneus

A BME near the tendon may reveal tendinopathy (Figs. 8 and 9), impingement (Figs. 10 and 11), tendon maltracking, or enthesopathy (Fig. 12) [5, 8, 12, 13]. Tendinopathy encompasses a wide range of tendon changes involving the internal structure of the tendon (Figs. 8 and 9), most commonly associated with micro-trauma and secondary degeneration. Changes in the bone marrow in direct contact with the tendon may indicate pathology of these tendons or tendon maltracking (Fig. 8). The presence of BME is probably due to altered tendon pressure on the bone [8, 12, 13]. Several authors report BME at the tendon insertion (Figs. 10, 11, and 12) to be a sign of enthesitis in inflammatory arthropathy [8, 14–16].

Reticular BME in the posterior half of the medial malleolus is seen with dysfunction of the tibialis posterior tendon (Fig. 8); BME in the posterior half of the lateral malleolus with pathology in the peroneal tendons (Fig. 9)—functional dysfunction, overuse, or ruptures [17, 18].

### Stress fracture

**Fig. 13:**
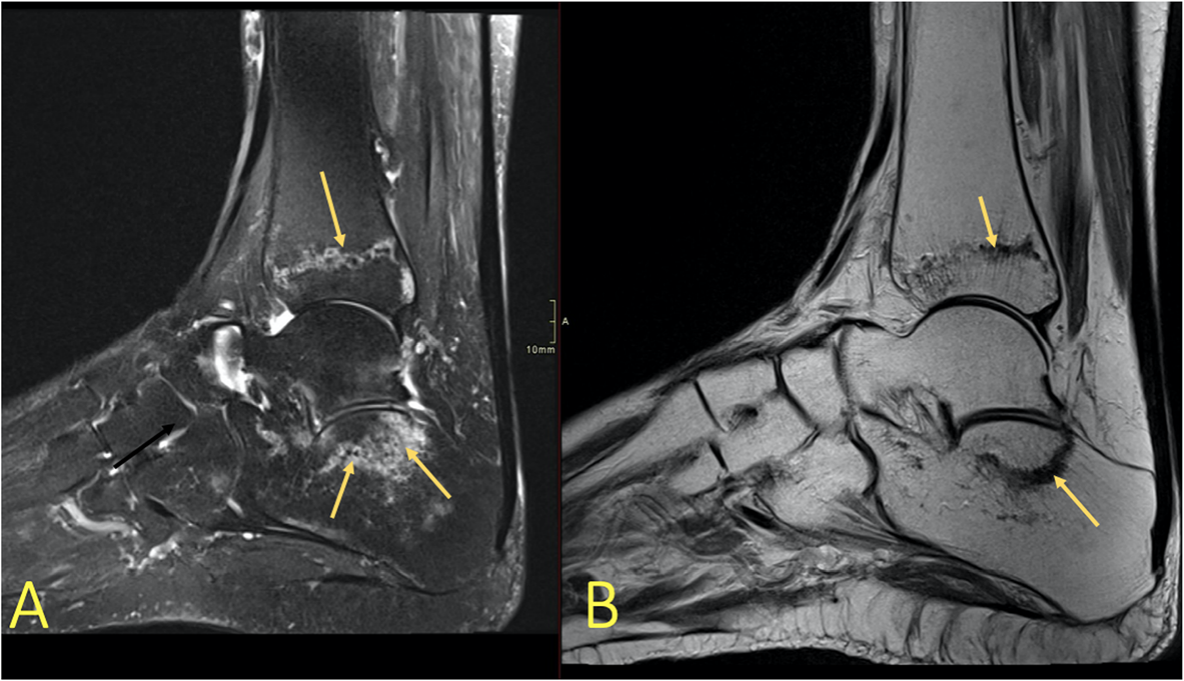
A 57-year-old female with hindfoot pain for 4 weeks and a suspected osteochondral lesion and osteoarthritis. MRI showed stress fractures of the tibia and calcaneus (arrows)

**Fig. 14:**
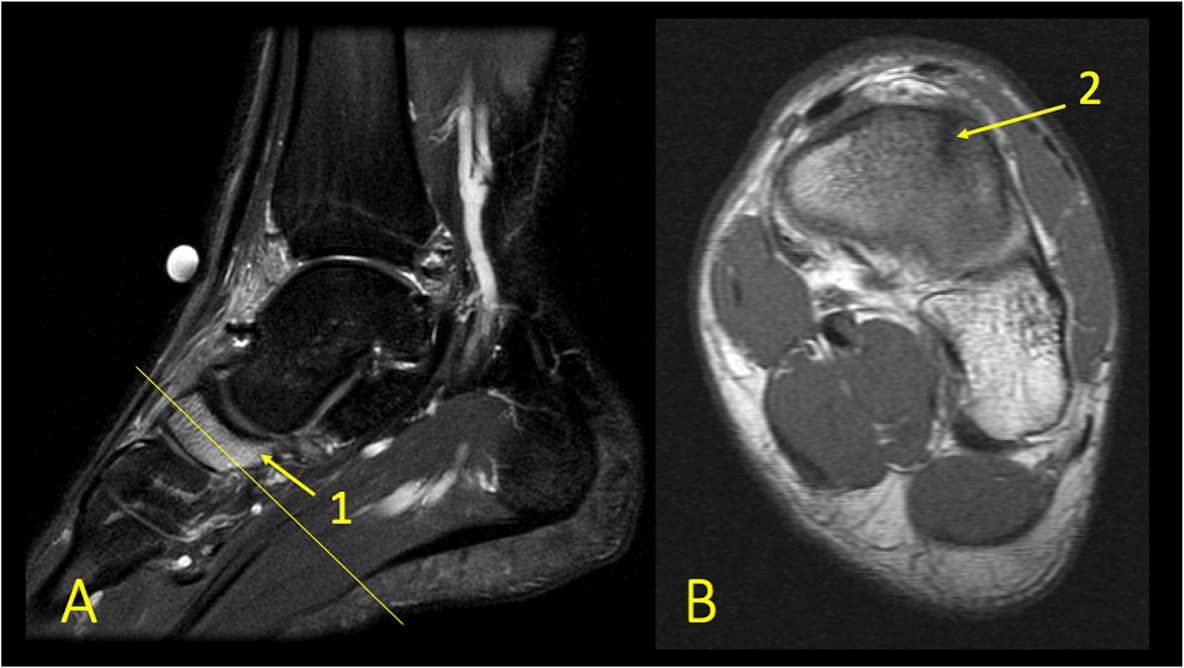
An 18-year-old female with diffuse pain at the level of the navicular bone. MRI with (**a**) T2-weighted fat suppression and (**b**) T1-weighted coronal images revealed extensive BME (1) and a stress fracture in the navicular bone (2)

When the bony trabecular load is higher than normal, loss of mechanical integrity through injury is possible. This happens with weakened bone tissue (osteopenia) or with vigorously repeated mechanical forces (Fig. 13). In these situations, a fatigue fracture or insufficiency fracture, respectively, may occur [5, 16]. Linear low-signal disturbances oriented perpendicular to the load axis, usually surrounded by extensive BME, are consistent with stress fracture (Fig. 14). A slight periosteal edema is the first sign of stress fracture which, in the absence of treatment, is followed by BME in the medullary cavity. Intracortical changes occur while the limb is still bearing weight. MRI can detect a stress fracture in the early stages [5, 8, 12, 13].

## Avascular necrosis and bone infarction

**Fig. 15:**
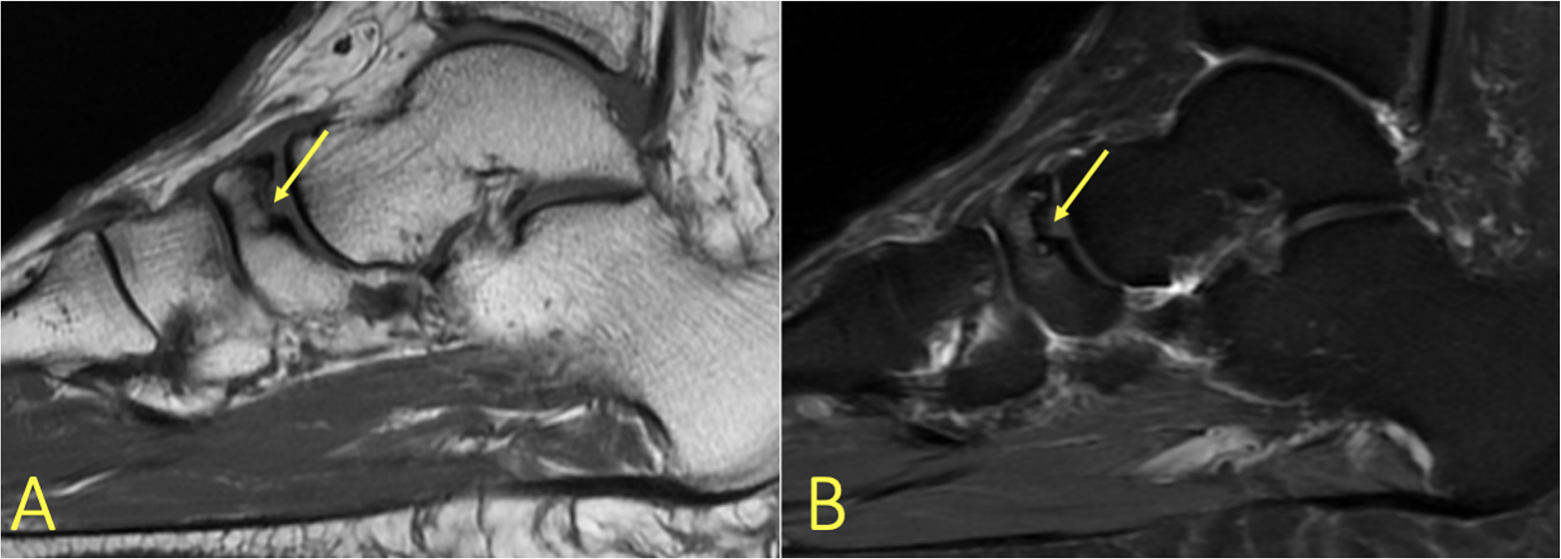
A 61-year-old female with hindfoot pain. MRI showed osteonecrosis of the navicular bone (arrows; Mueller-Weiss syndrome)

**Fig. 16:**
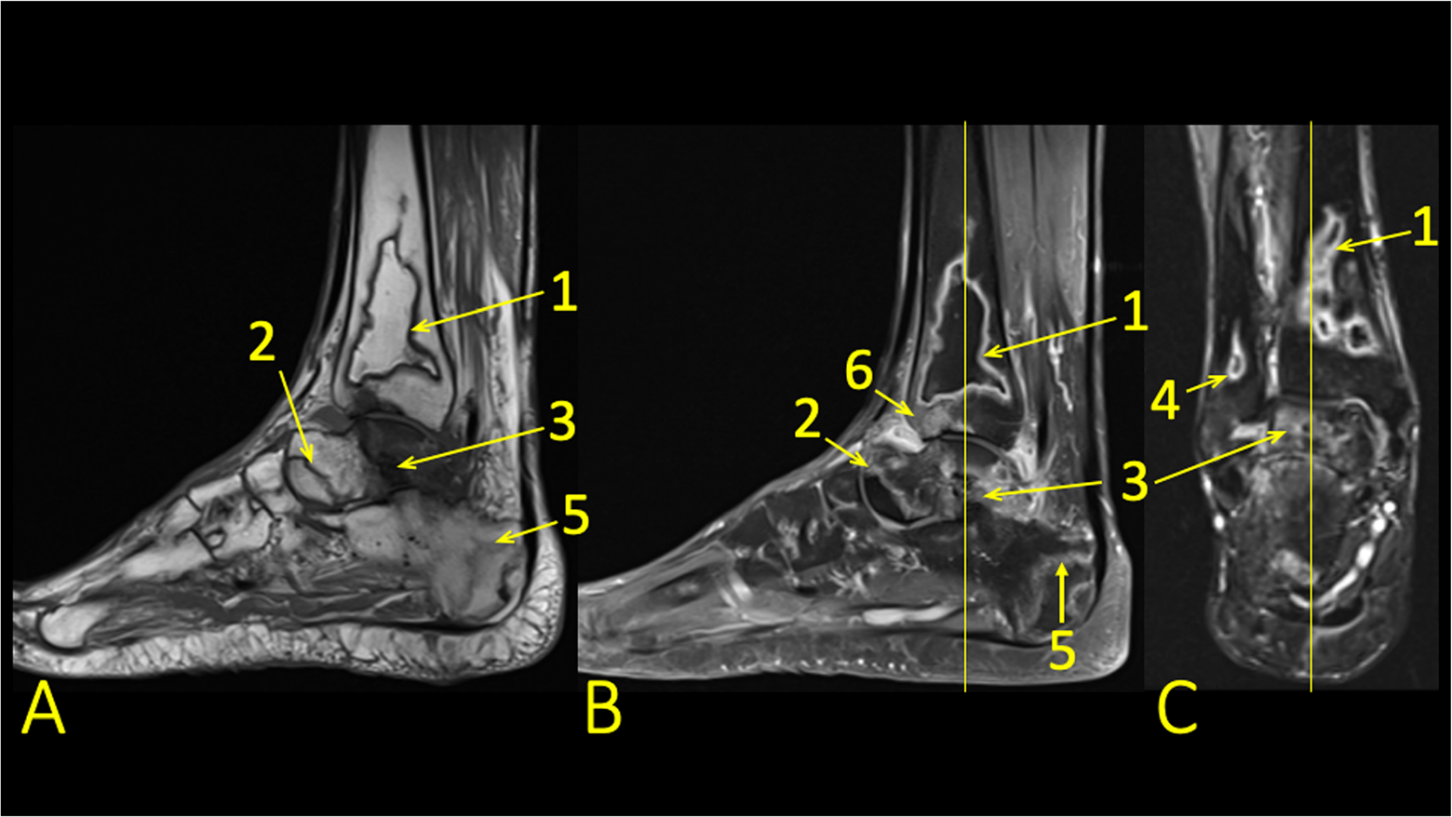
A 45-year-old male, 2 years after kidney transplantation, presented with 6-week history of ankle pain and a suspected talar stress fracture. MRI showed bone infarcts in the distal tibia (1), fibula (4), talus (2), and calcaneus (5) and a talar fracture (3). BME in the subchondral part of the distal tibia (6)

Avascular necrosis and bone infarction can be a complication of fracture, most often seen in the talus, followed by the navicular bone (Fig. 15) and the fifth metatarsal bone. Bone infarction may also result from inflammatory, metabolic, or genetic diseases such as systemic lupus erythematosus or sickle-cell disease or may be a side-effect of medical treatment with, e.g., steroids. A bone marrow signal alteration in the form of a serpentine line indicates a bone infarct [9, 11]. On T2-weighted images, this line usually comprises two components; a low signal located internally and a high signal externally. These correspond to infarct and hyperemia, respectively, called a double line sign (Fig. 16).

## Cartilage and subchondral bone

**Fig. 17:**
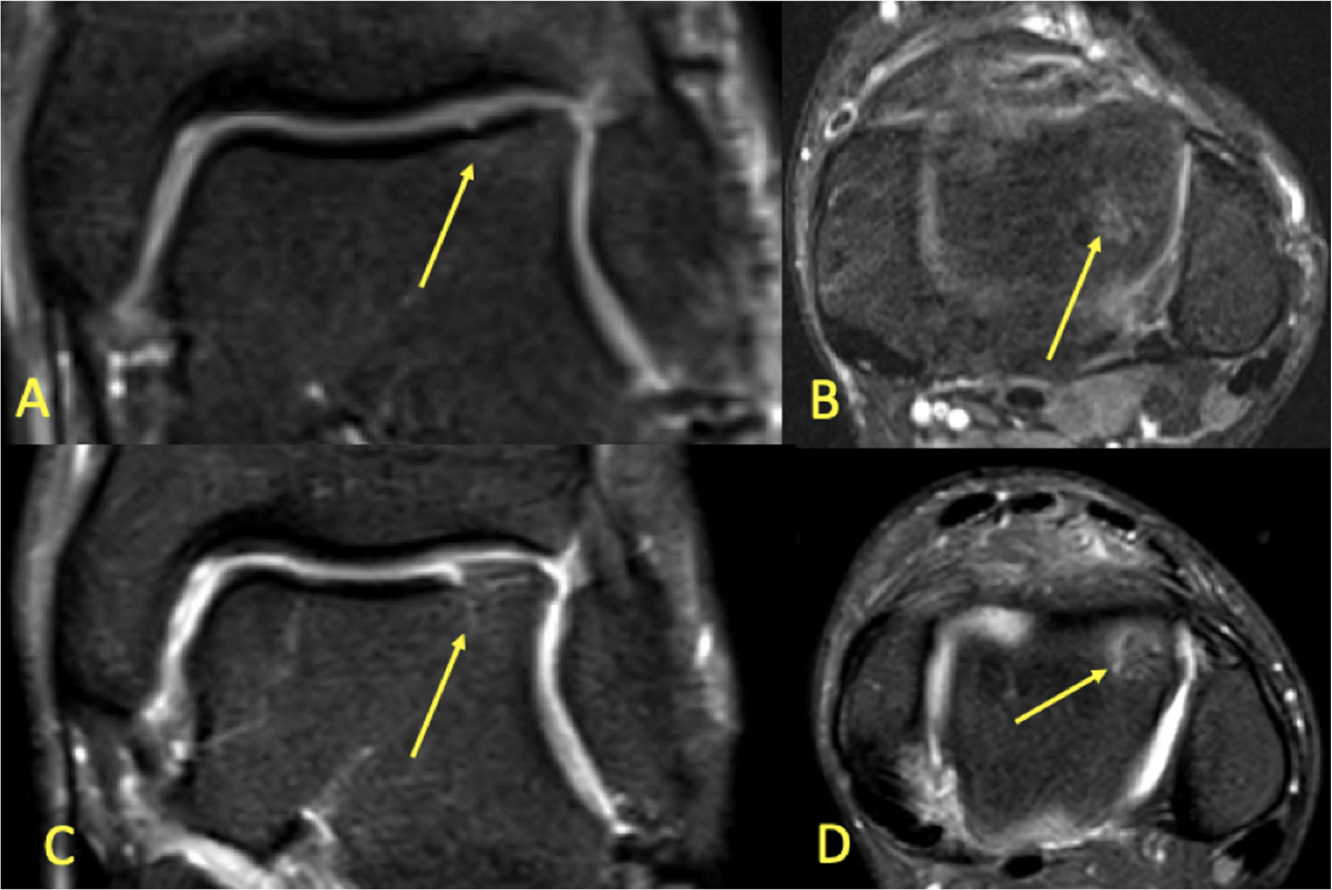
A 19-year-old handball player with 3-month history of ankle pain imaged for suspected anterior tibiofibular ligament rupture and stress fracture. **a**, **b** On the initial MRI, the cartilage lesion (arrow) was missed, and neither an anterior tibiofibular ligament rupture nor a stress fracture was detected. **c**, **d** Because of persistent pain preventing training, MRI was repeated after 8 months. A chondral lesion with subchondral BME was visible and could be identified retrospectively on the previous MRI (arrows)

**Fig. 18:**
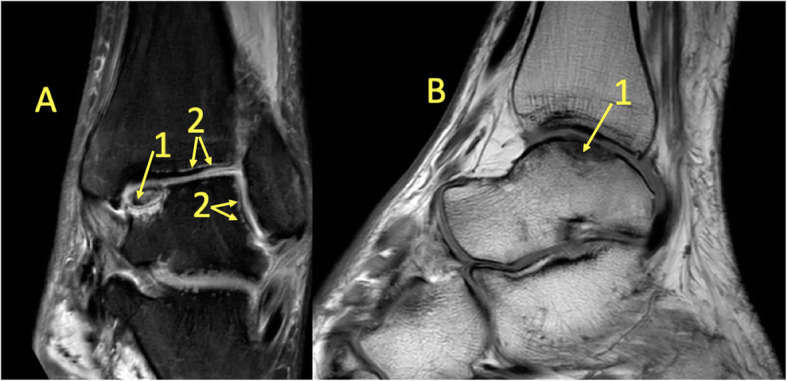
A 29-year-old male with 4-month history of medial ankle pain and suspicion of an osteochondral lesion. (1) MRI showed a stable non-displaced osteochondral lesion and (2) subchondral BME, consistent with early inactive osteopenia

Degenerative changes or minor cartilage structural damage with a changed load on the subchondral layers may cause subchondral BME [7, 8, 11, 12]. Often, relatively minor changes in the signal of the subchondral bone marrow can indicate relatively large cartilage lesions. The reason is that the thin cartilage layer is difficult to image on MRI. An altered subchondral layer signal may help with cartilage assessment (Fig. 17c, d). Progression of subchondral BME is usually associated with the progression of cartilage damage, typically in the talar trochlea and distal tibia. An osteochondral lesion covers both the cartilaginous and subchondral layers (Fig. 18). An unstable osteochondral lesion is a bone fragment which loses contact, displaces, or separates from the bone into the surrounding joint fluid [8, 19]. Osteochondritis dissecans (OCD) may be loose in situ if there is surrounding BME [3, 8]. If untreated, it may cause subchondral damage (Fig. 17a, b) [5].

### Impingement

BME can be a very sensitive sign in the different types of impingement [11, 20]. Posttraumatic changes such as local synovitis, scars, thickened articular capsule or ligaments, and osteophytes may cause different impingement syndromes based on the localization. The most common impingement is anterolateral impingement, followed by posterior and anterior impingement. BME is a bone manifestation of impingement [17, 20].

Anterolateral impingement is an uncommon complication of ATFL injury resulting in hemarthrosis and synovitis, less often in the thick inferior part of the ATFL. The anterolateral gutter becomes filled with synovitis, scar tissue, and small osseous fragments [20].

An elongated posterior process of the talus or an os trigonum causes posterior ankle impingement. BME found in the posterior process of the talus or in the os trigonum, edema in Kager’s fat pad, and effusion in the posterior ankle recess are frequent findings [20, 21].

**Fig. 19:**
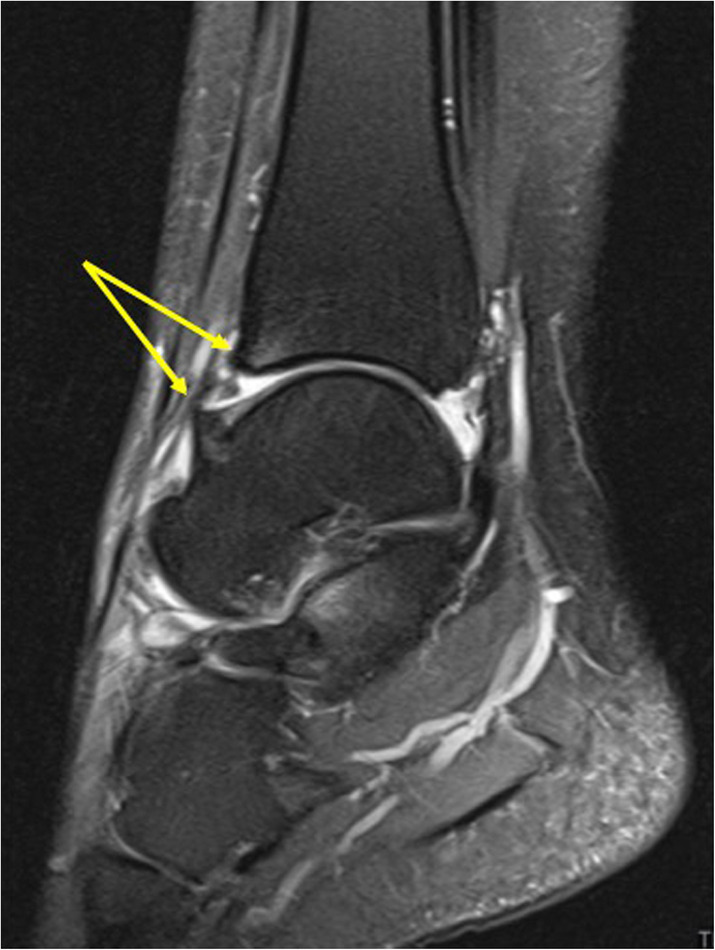
A 22-year-old soccer player presented with chronic anterior ankle pain. There was no history of specific ankle trauma leading to suspicion of anterior impingement. MRI showed anterior impingement with osteophytes (arrows) and bone marrow edema

**Fig. 20:**
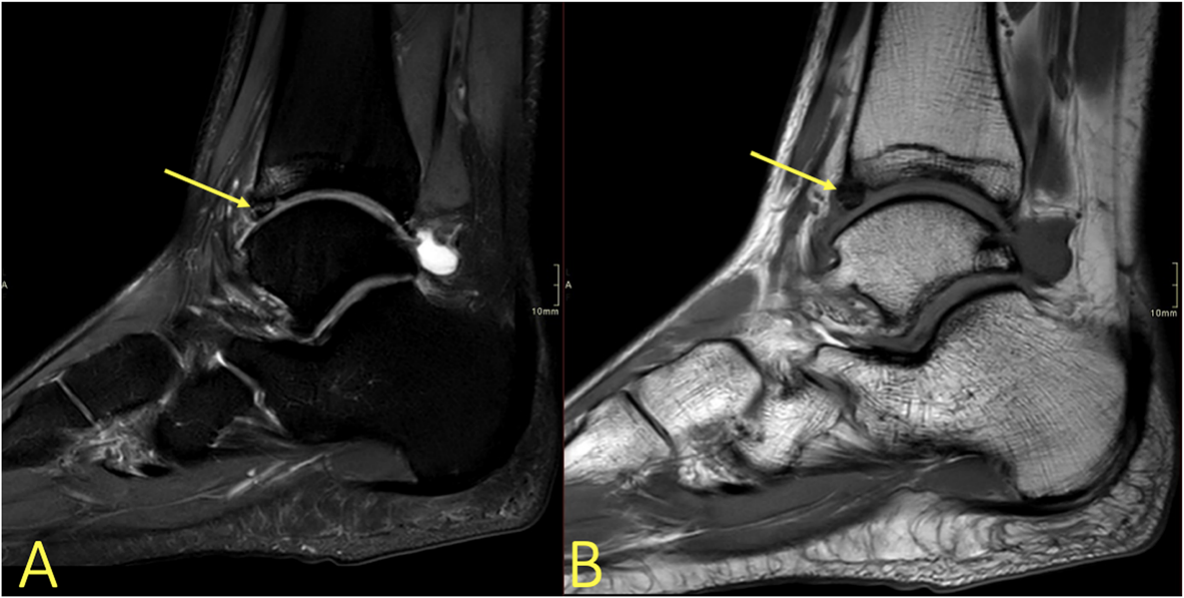
A 55-year-old male with chronic anterior ankle pain. He had had a previous ankle injury about 8 years ago without fracture but now suspicion of developing osteoarthritis. MRI showed osteophytes at the anterior border of the distal tibia with bone marrow edema (arrows) showing anterior impingement

Anterior impingement manifests by a painful limitation of dorsal ankle flexion, mostly in football players, called “footballers’ ankle.” Accumulation of micro-trauma on the talar neck and anterior distal tibia may results in synovitis, thickening of the articular capsule, or osteophytes with BME (Figs. 19 and 20) [17, 20].

**Fig. 21:**
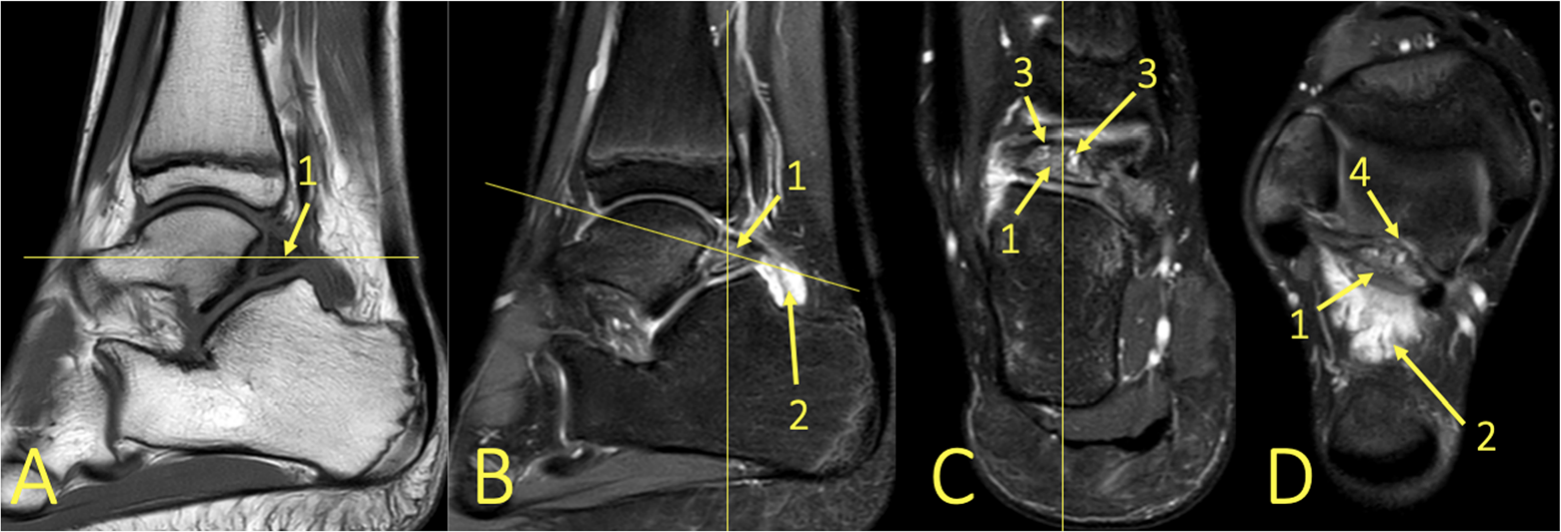
A 16-year-old male with chronic posterior ankle pain and a suspected os trigonum with impingement. (1) MRI showed an os trigonum with BME, (2) effusion and synovitis, and (3) degenerative cysts (4) along the articulation with the posterior talar process

An evaluation of BME allows for better identification of patients for whom surgery is indicated (Figs. 19, 20, and 21).

### Accessory bones

**Fig. 22:**
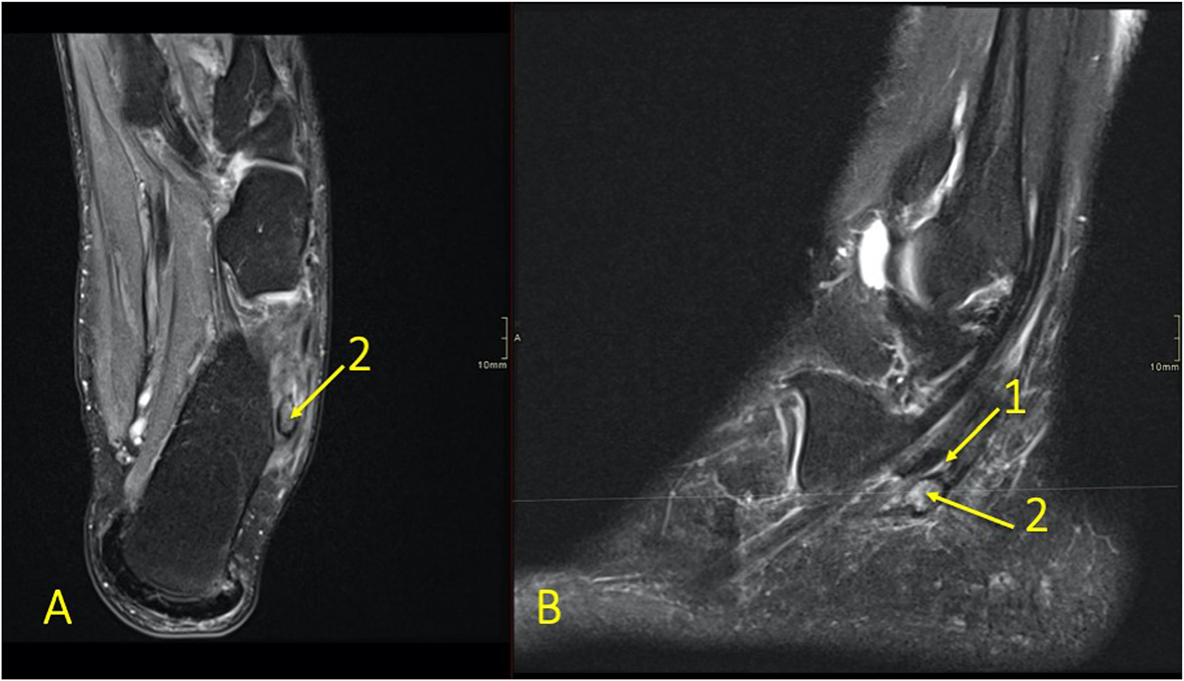
A 32-year-old handball player presented after a match with lateral ankle pain and a suspected lateral malleolar fracture. (1) MRI revealed a split rupture of the peroneus longus tendon (2) at the level of an os peroneum with BME

BME in an accessory bone shows that it is probably a symptomatic accessory bone. Accessory bones occur at various frequencies but rarely cause discomfort and are often bilateral. The most frequent problem they cause is being mistaken for a fracture. The most common accessory bones are the os peroneum, os naviculare accessorium, and os trigonum. The os trigonum and talus often articulate. The mobility produces a folding of the joint capsule, occurrence of joint fluid, and conflict with the synovial sheath of the flexor hallucis longus tendon or Kager’s fat pad. BME usually occurs both in the os trigonum and the adjacent part of the talus (Fig. 21) if it is symptomatic [21]. An accessory bone in a tendon (e.g., os peroneum) may cause tendon overload and faster tendon degeneration and rupture (Fig. 22).

### Tarsal coalition

**Fig. 23:**
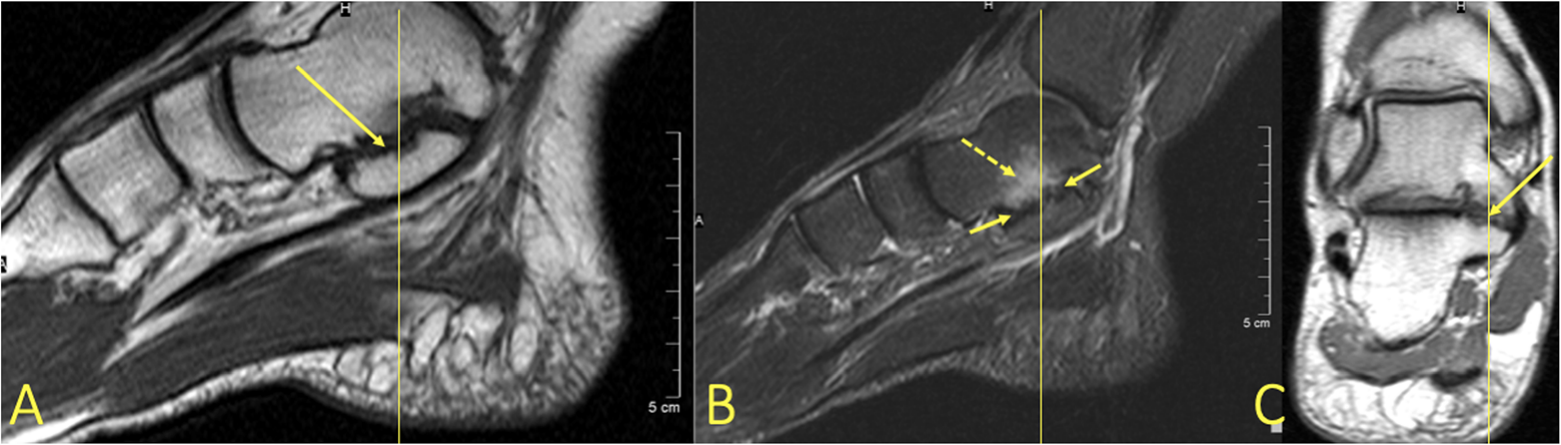
A 43-year-old female with chronic ankle pain and suspicion of an osteochondral lesion and osteoarthritis. MRI showed a talocalcaneal coalition (arrow) with BME in the talus (dashed arrow)

**Fig. 24:**
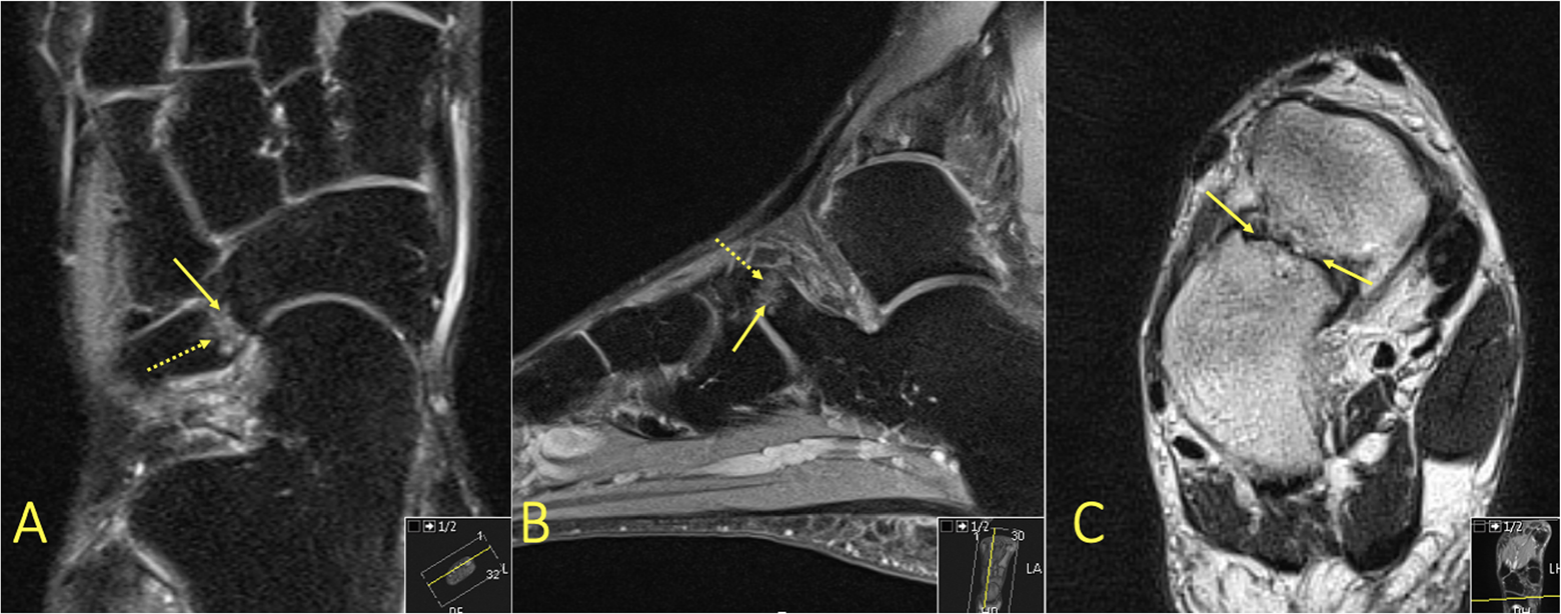
A 51-year-old female with diffuse pain in the ankle and Chopart joints with suspected osteoarthritis. MRI showed a calcaneonavicular coalition (arrows) with adjacent BME (dashed arrow)

A coalition is an abnormal developmental fusion between bones. It usually results from the failure of segmentation of the bones during development, which in the foot occurs in about 1% of the population [22, 23]. A coalition may be bony or fibrous. The coalition prevents proper movement in the joint, which causes deformity, pain, and soft tissue changes. The distortion in fibrous coalition results in overuse and subsequent BME. Two of the most common coalitions are between the calcaneus and the navicular bone or between the middle facet of the talus and the calcaneus [22, 24] (Figs. 23 and 24).

## BME not associated with trauma

**Fig. 25:**
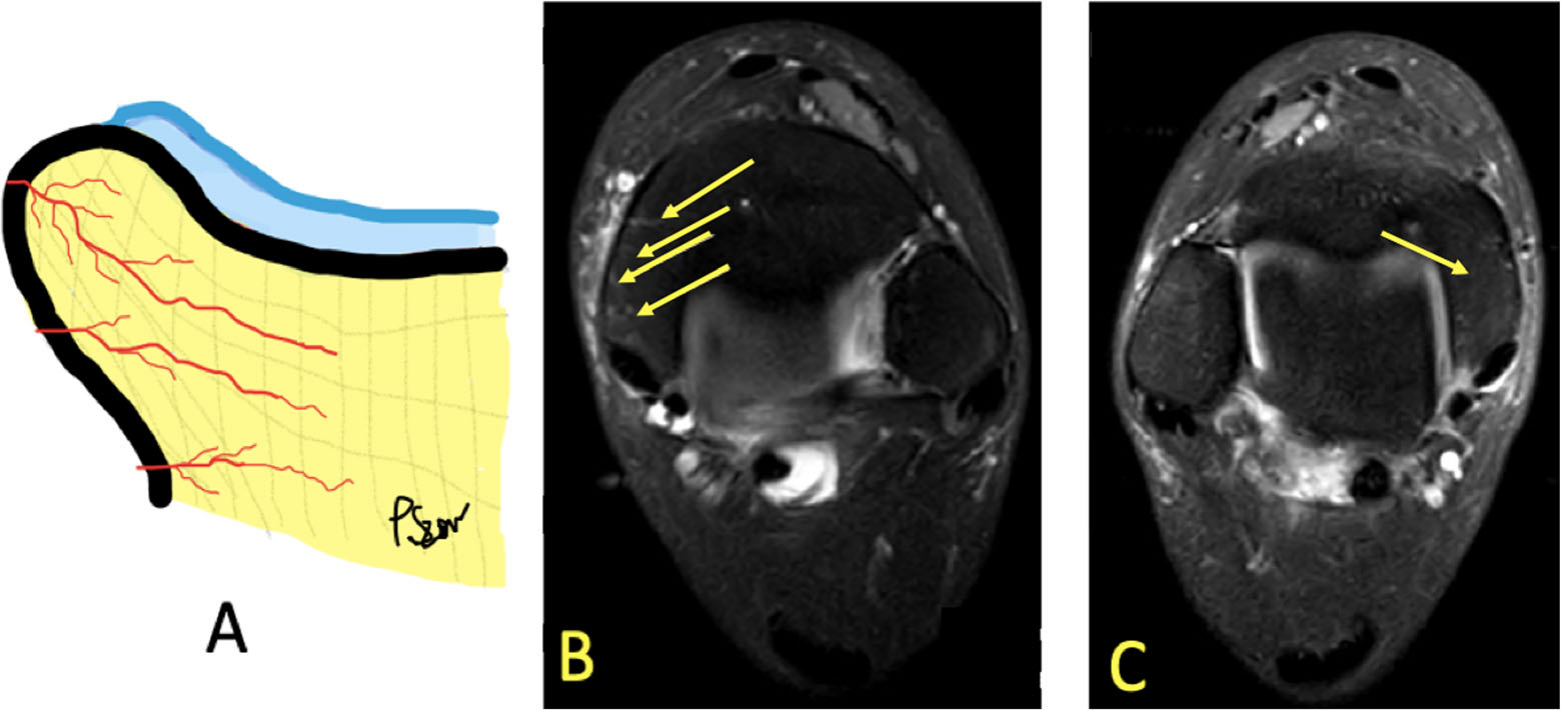
A 32-year-old runner with 1-month history of right medial ankle pain and suspected stress fracture of the medial malleolus. **a** Drawing of blood vessels in the bone marrow. **b** MRI showed hyperemia of bone marrow in the medial malleolus on the symptomatic side (arrows). **c** On the contralateral side, no vessels were visualized (arrow)

BME may occur in physically active individuals. The cause, however, is not fully understood [1, 9, 25]. It is probably a consequence of microscopic changes in the bone, like bone remodeling and micro-fractures of the bony trabeculae [2, 9]. Hyperemia of the bone marrow (Fig. 25) related to a weight-bearing surface, a physiologic process, is often visible [9].

### Infectious and inflammatory processes

**Fig. 26:**
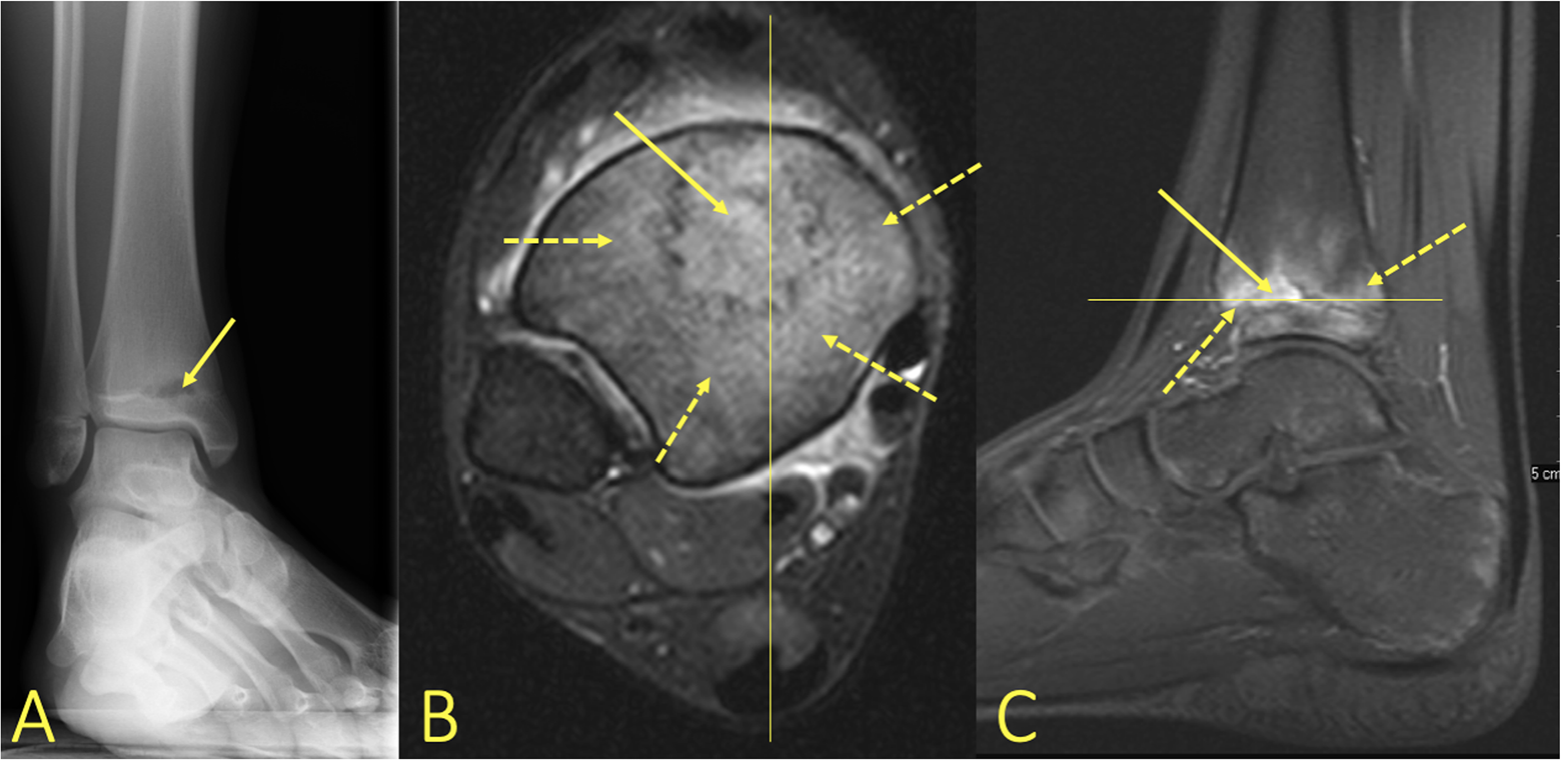
A 13-year-old female with intermittent moderate ankle pain for 3 weeks. **a** Radiographs showed a well-defined radiolucent lesion centrally in the distal tibial metaphysis (arrow). **b**, **c** MRI showed a Brodie abscess (arrow) with BME (dashed arrows) and contrast enhancement in distal tibia (arrow)

**Fig. 27:**
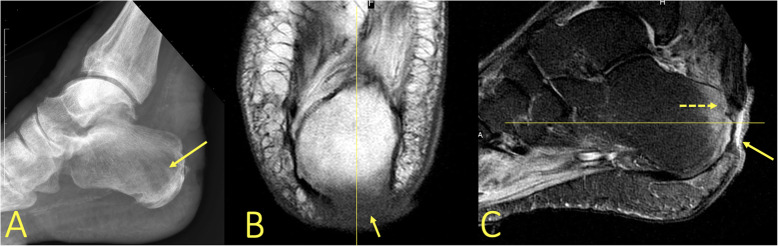
A 66-year-old male with diabetes. **a** Osteomyelitis in calcaneus was suspected clinically and on radiographs. **b**, **c** MRI confirmed the diagnosis (arrows), BME (dashed arrow)

**Fig. 28:**
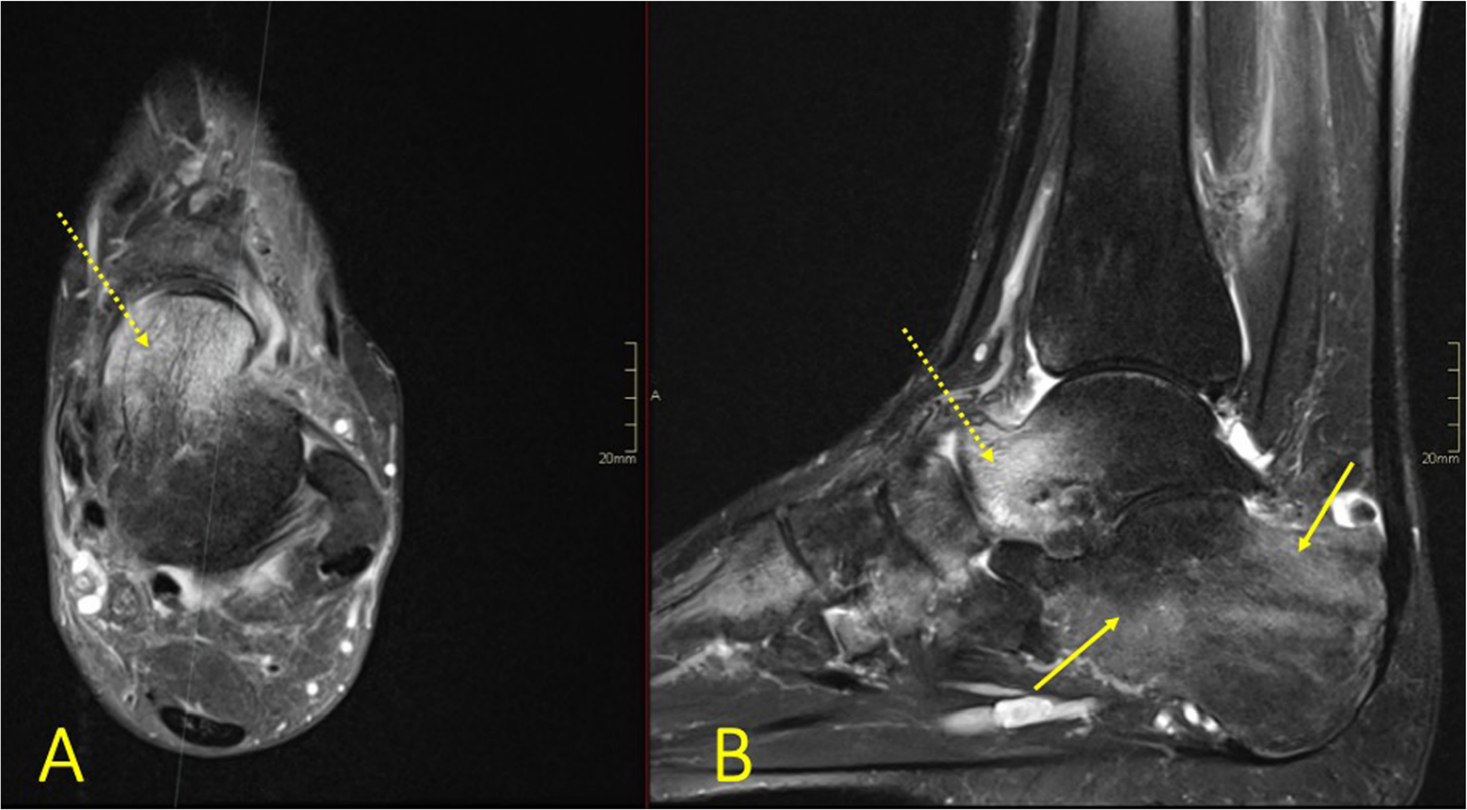
A 70-year-old female with diabetes and Charcot foot. MRI showed extensive bone marrow edema (dashed arrows) in all bones of the foot suggesting active Charcot changes, most prominent at the level of the Lisfranc joint, where dorsal dislocation of os cuneiforme intermedium is visible (arrow)

BME is common in patients with septic arthritis and osteomyelitis. In areas of bone with inflammation, fluid signal replaces the fat signal. Usually, the fluid shows a higher signal than usual on T1-weighted images because of an increased protein concentration [8]. In osteomyelitis, the geographic replacement of the fat signal on T1-weighted images by edematous low signal has been reported to correspond better to the distribution of infection (Figs. 26 and 27) than the more diffuse reticular BME seen on fluid-sensitive images [26]. Similar imaging findings can be seen in chronic recurrent multifocal osteomyelitis (CRMO) where, however, no pathogenic agent has been confirmed (Fig. 28) [27]. BME is suggestive rather than pathognomonic for CRMO. In CRMO, a transient BME manifests as a region of BME without trauma (Fig. 28), which usually resolves in 3–12 months. The pathogenesis is not clear, and both a vascular and a neurologic etiology have been proposed [2, 4, 7, 28].

In inflammatory arthritides like rheumatoid arthritis (RA) or spondyloarthritis, hindfoot pain is not uncommon and is especially in spondyloarthritis an important diagnostic component if caused by enthesopathy with BME. Often, synovitis and joint effusion causes the pain [29]. Retrocalcaneal bursitis may lead to BME [30, 31]. BME in RA, known as osteitis and most commonly investigated in the hand and forefoot, is associated with erosive progression and poor functional outcome [32]. In spondyloarthritis, many patients, even if asymptomatic, have BME and other arthritis-related changes [30, 33].

**Fig. 29:**
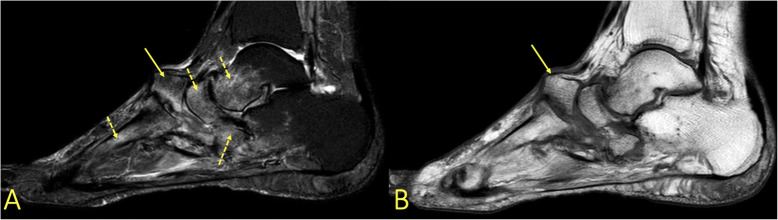
A 28-year-old male presented with ankle pain for 3 months without a history of trauma and suspicion of tumor. There was extensive bone marrow edema in all bones of the foot, most pronounced in the talus (dashed arrow on **a**) and calcaneus (arrow on **b**). CRMO was diagnosed after a couple of months

BME with associated soft tissue edema in patients with peripheral neuropathy can indicate neuropathic arthropathy (Fig. 29) [11, 26]. The presence of BME in the early stages shows the extent of the process, while in the later stages, it helps distinguish the vital areas from osteonecrosis [34].

## Perilesional reaction

**Fig. 30:**
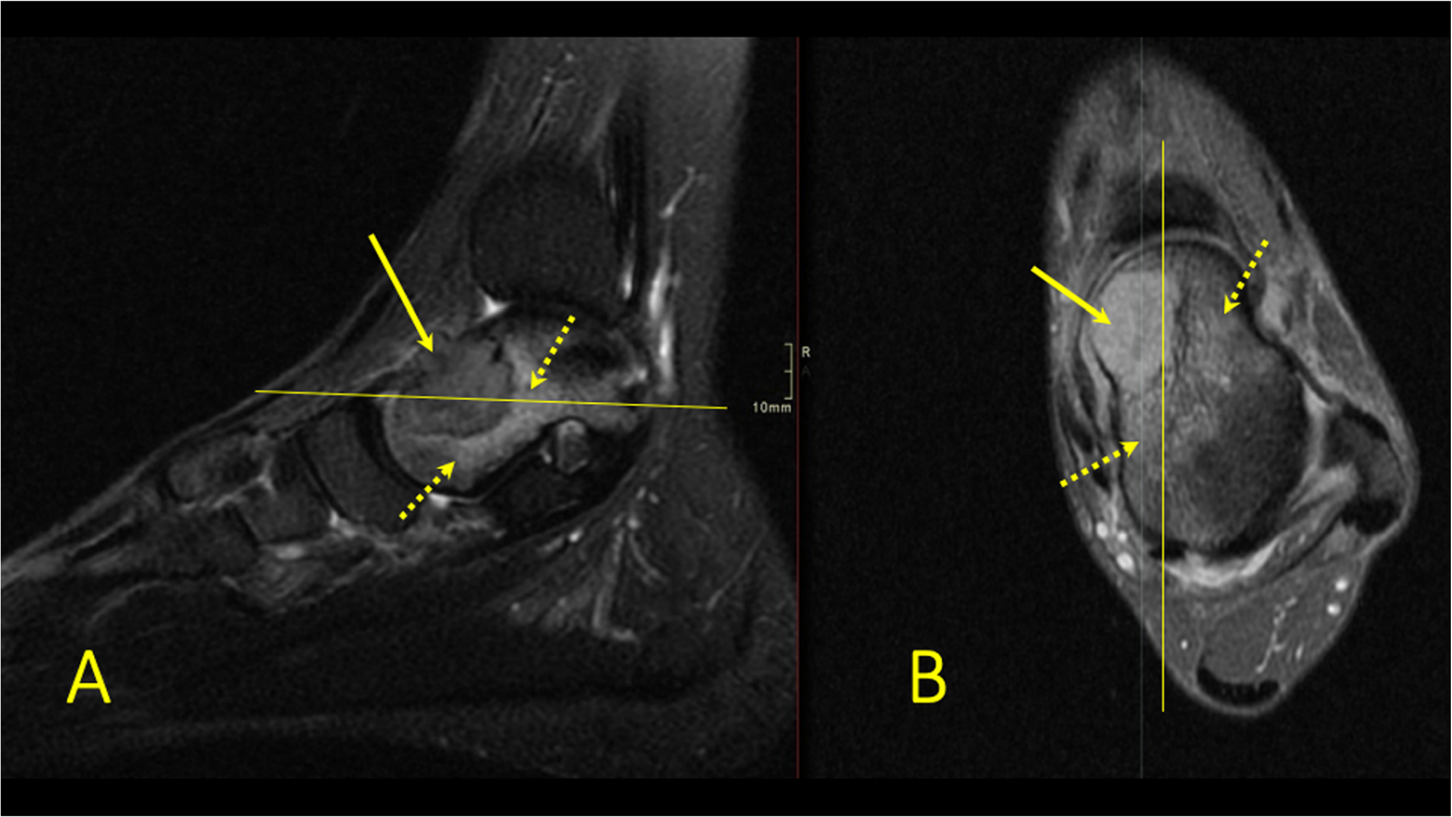
A 28-year-old male presented with 3-month history of ankle pain and a suspected osteochondral lesion. MRI showed a solid lesion in the talar head and neck (arrow) with BME as a perilesional reaction (dashed arrow). Histological diagnosis was giant cell tumor

**Fig. 31:**
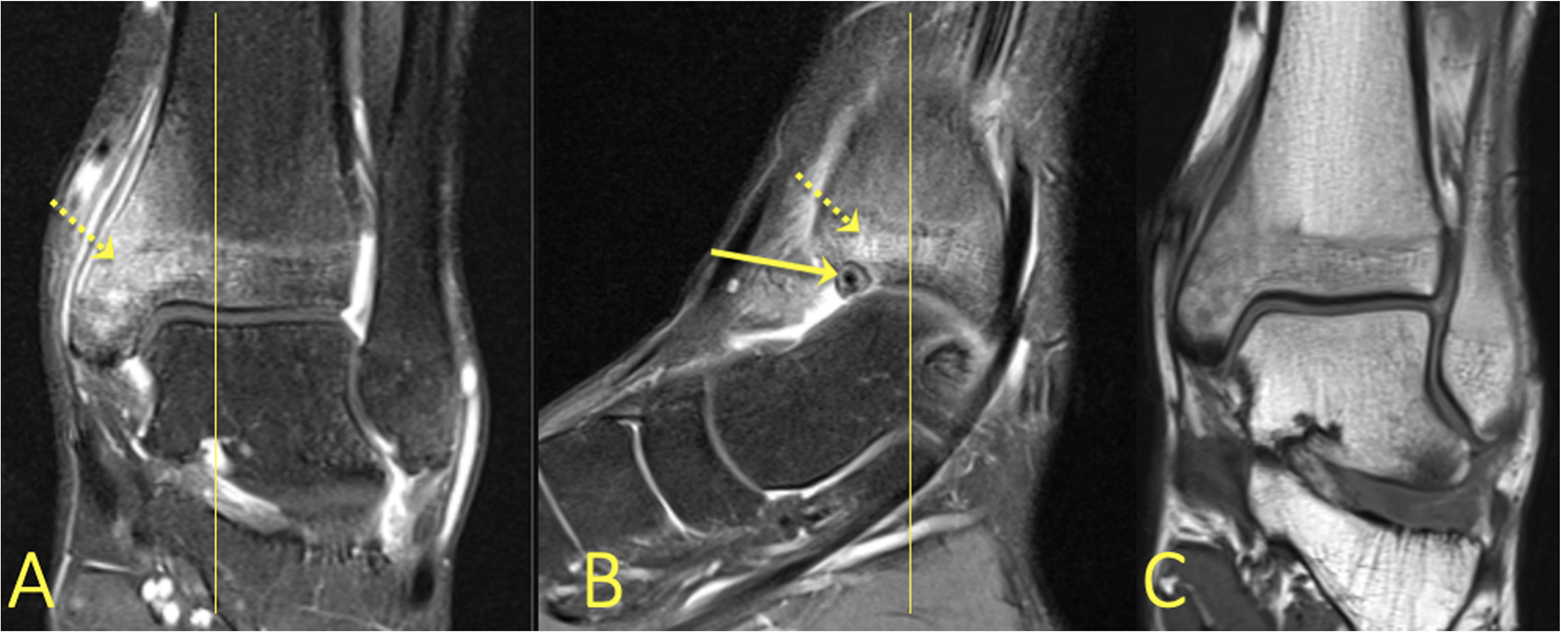
A 19-year-old male presented with nightly ankle pain. MRI confirmed a suspected osteoid osteoma, showing an intra-articular osteoid osteoma (arrow) with minimal sclerosis and extensive BME (dashed arrows) as a perilesional reaction

A perilesional reaction with perilesional BME sometimes surrounds a focal lesion (Fig. 30). Local trabecular destruction and direct physical insult with neovascularization and inflammation is the background for tumor-induced BME [3, 6, 35]. That is the reason a contrast agent is usually required to differentiate a tumor from BME [35]. Both benign and malignant tumors can be associated with BME. The most common benign tumors are osteoid osteoma (Fig. 31), osteoblastoma, and chondroblastoma. BME usually surrounds both primary malignant bone tumors (like osteosarcoma, Ewing’s sarcoma, or chondrosarcoma) and metastases [35].

**Fig. 32:**
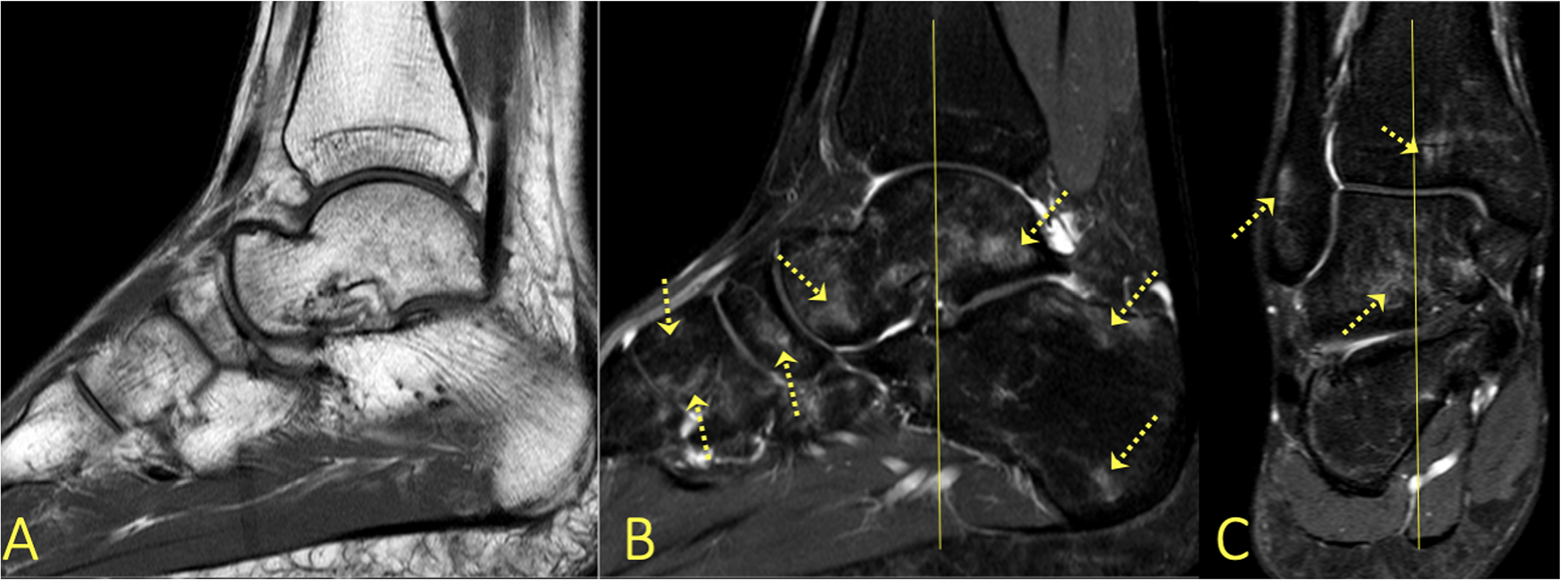
A 21-year-old male with inactive osteopenia after immobilization for some weeks. Patchy lesions are visible in all bones (dashed arrows)

BME appears after immobilization, sooner in younger than in older individuals [1]. The alteration of the bone marrow signal is located mostly peripherally and has a patchy appearance (Fig. 32); the patient’s history confirms the interpretation. Disorders in the autonomic innervation of vessels are the main background for BME after immobilization [1, 9].

## Pitfalls

**Fig. 33:**
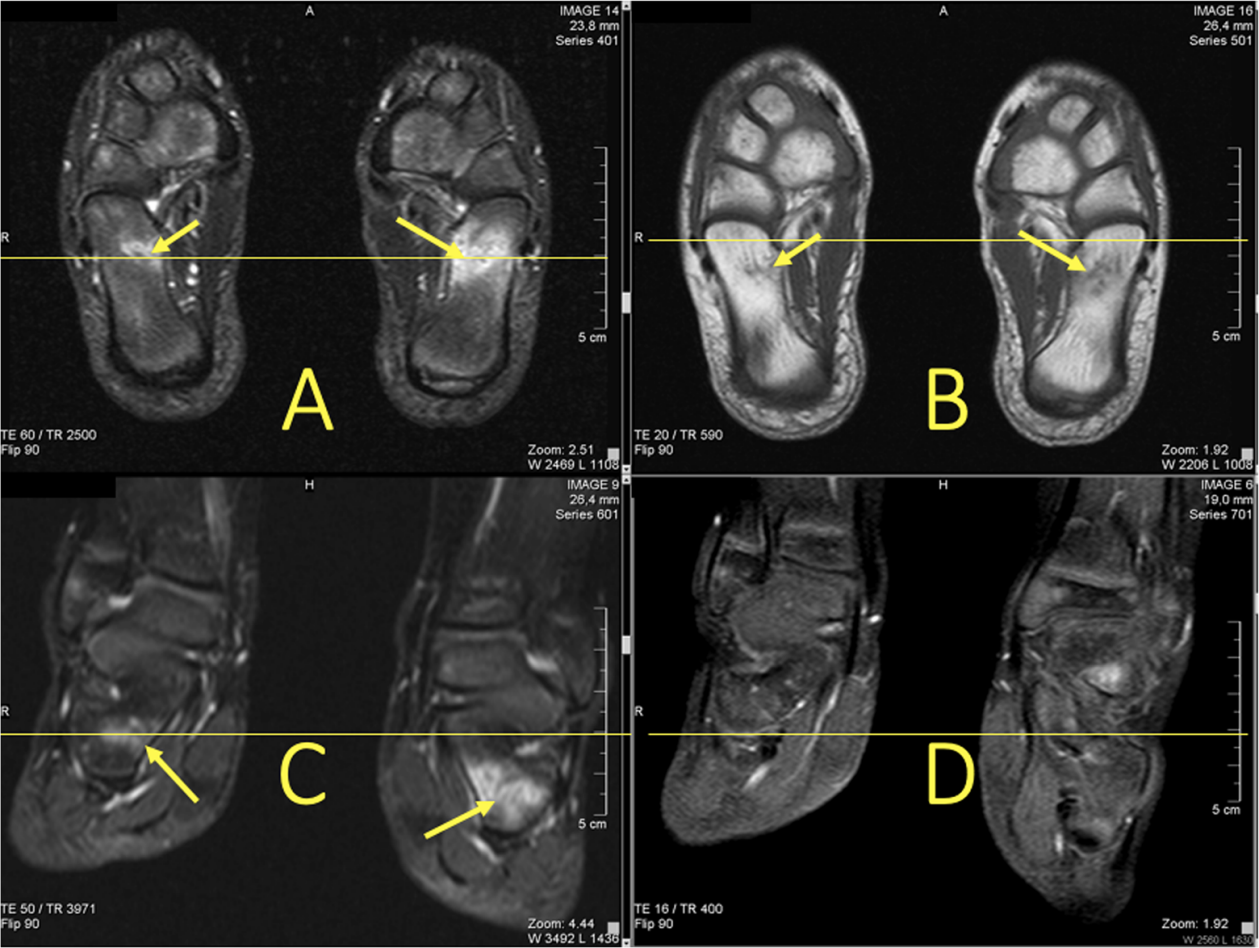
A 7-year-old girl with chronic pain in both feet. MRI shows a developmental variation in the bone marrow signal in the calcaneus (arrows)

Remnants of red bone marrow in growing patients may resemble BME but is a normal finding in typical localizations (Fig. 33). An edema-like high signal may also be related to physiologic stress or altered biomechanics in the growing skeleton [36], as well as in highly active individuals. There are edema features that can differentiate red bone marrow from BME, like a lower signal, localization in the metaphysis, or well-defined margins [7, 19].

**Fig. 34:**
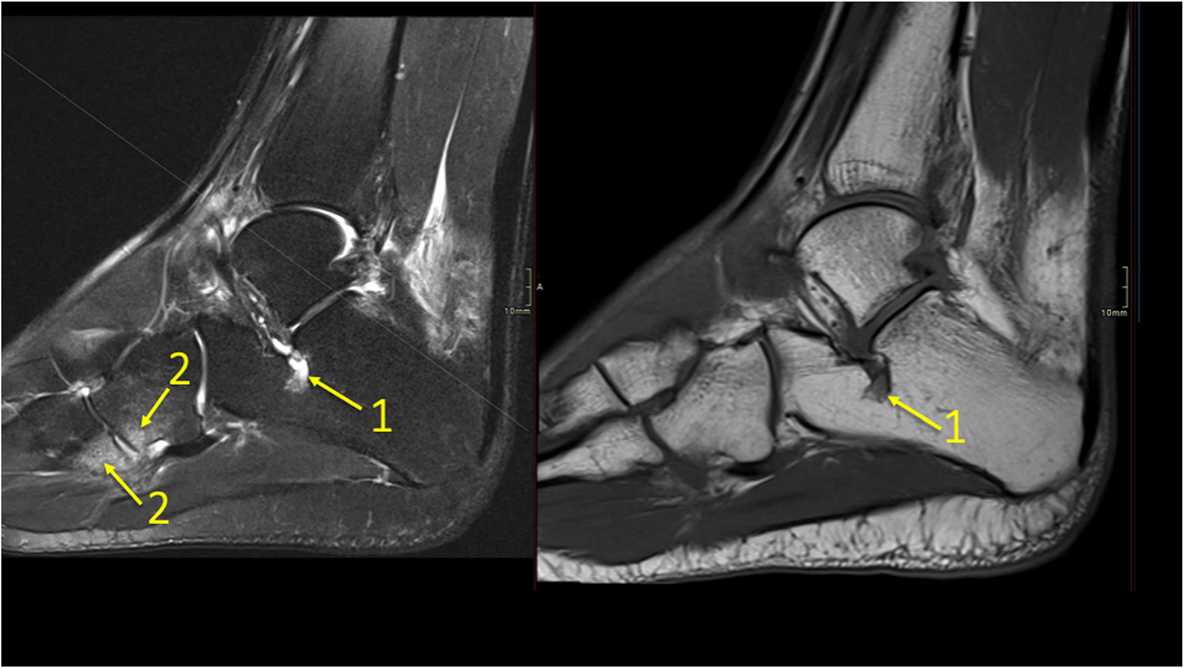
A 30-year old patient after ankle joint sprain. Radiographs revealed a Lisfranc fracture-dislocation. (1) MRI showed a vascular remnant, a pitfall, in the calcaneus. (2) BME was seen at the level of the Lisfranc joint

In the region of the sinus tarsi in the calcaneus, a cystic structure is often observed which is a vascular remnant (Fig. 34).

**Fig. 35:**
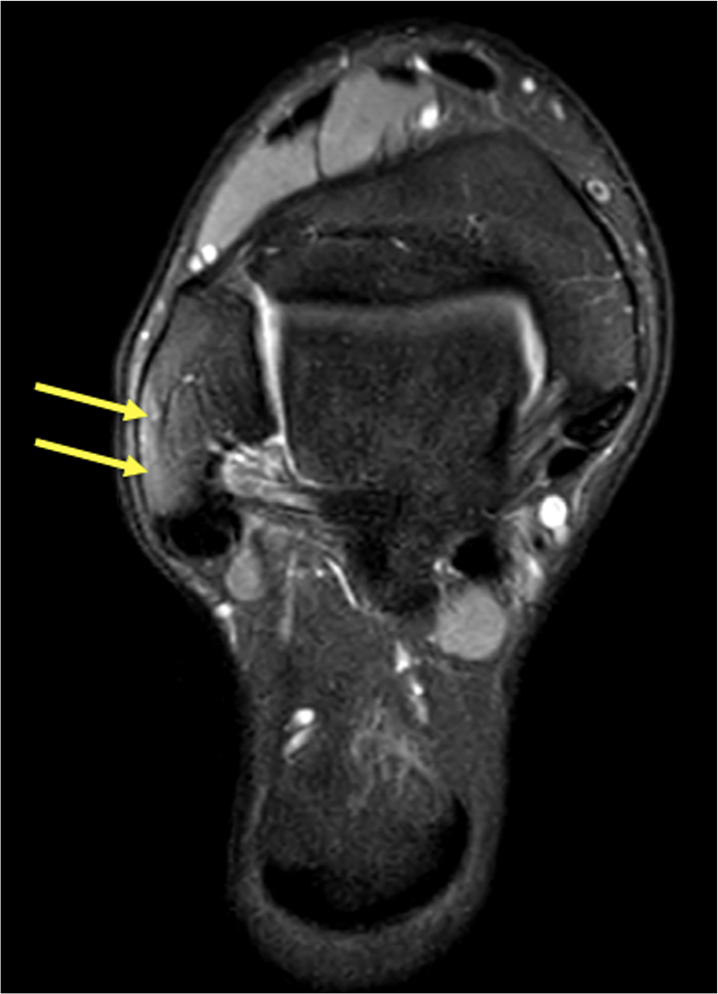
An 18-year-old runner with Achilles tendon pain. MRI showed a typical artifact in the lateral malleolus on an FS sequence (arrows). There were no changes in the Achilles tendon

In the ankle region, MR artifacts are typically located at the level of the lateral malleolus and may imitate BME. A higher signal of the bone marrow in the distal fibula on fluid-sensitive sequences is related to a close location to the coil (Fig. 35).

## Conclusion

The distribution of BME seen in specific types of injury represents one of the most useful differential diagnostic clues in ankle MRI. A systematic BME analysis allows for correct interpretation, which enables a correct assessment of the entire MR study. Configurations of BME act as footprints in trauma and help in an assessment and diagnosis of musculoskeletal injuries, prompting the radiologist to assess structures with pathologic findings which can significantly influence patient treatment.

## Data Availability

Yes

## Abbreviations

BME: Bone marrow edema
BML: Bone marrow lesion
FS: Fat-suppressed
MRI: Magnetic resonance imaging
PD: Proton-density
STIR: Short tau inversion-recovery

## References

[1] Elias I, Zoga AC, Schweitzer ME, Ballehr L, Morrison WB, Raikin SM (2007). A specific bone marrow edema around the foot and ankle following trauma and immobilization therapy: pattern description and potential clinical relevance. Foot Ankle Int.

[2] Patel CV (2009). The foot and ankle: MR imaging of uniquely pediatric disorders. Magn Reson Imaging Clin N Am.

[3] Sijbrandij ES, van Gils APG, de Lange EE (2002). Overuse and sports-related injuries of the ankle and hind foot: MR imaging findings. Eur J Radiol.

[4] Orr JD, Sabesan V, Major N, Nunley J (2010). Painful bone marrow edema syndrome of the foot and ankle. Foot Ankle Int.

[5] Vanhoenacker FM, Snoeckx A (2007). Bone marrow edema in sports: general concepts. Eur J Radiol.

[6] Eustace S, Keogh C, Blake M, Ward RJ, Oder PD, Dimasi al M (2001) MR imaging of bone oedema: mechanisms and interpretation. Clin Radiol 56:4–12. 10.1053/crad.2000.0585 10.1053/crad.2000.0585 11162690

[7] Fowkes LA, Toms AP (2010). Bone marrow oedema of the knee. Knee.

[8] Weishaupt D, Schweitzer ME (2002). MR imaging of the foot and ankle: patterns of bone marrow signal abnormalities. Eur Radiol.

[9] Rios AM, Rosenberg ZS, Bencardino JT, Rodrigo SP, Theran SG (2011). Bone marrow edema patterns in the ankle and hindfoot: distinguishing MRI features. AJR Am J Roentgenol.

[10] Costa-Paz M, Muscolo DL, Ayerza M, Makino A, Aponte-Tinao L (2001). Magnetic resonance imaging follow-up study of bone bruises associated with anterior cruciate ligament ruptures. Arthroscopy.

[11] Starr AM, Wessely MA, Albastaki U, Pierre-Jerome C, Kettner NW (2008). Bone marrow edema: pathophysiology, differential diagnosis, and imaging. Acta Radiol.

[12] Ahn JM, El-Khoury GY (2007). Role of magnetic resonance imaging in musculoskeletal trauma. Top Magn Reson Imaging.

[13] Niva MH, Sormaala MJ, Kiuru MJ, Haataja R, Ahovuo JA, Pihlajamaki HK (2007). Bone stress injuries of the ankle and foot: an 86-month magnetic resonance imaging-based study of physically active young adults. Am J Sports Med.

[14] McGonagle D, Gibbon W, Emery P (1998). Classification of inflammatory arthritis by enthesitis. Lancet.

[15] Rudwaleit M, van der Heijde D, Landewé R (2011). The Assessment of SpondyloArthritis International Society classification criteria for peripheral spondyloarthritis and for spondyloarthritis in general. Ann Rheum Dis.

[16] Kozoriz MG, Grebenyuk J, Andrews G, Forster BB (2012). Evaluating bone marrow oedema patterns in musculoskeletal injury. Br J Sports Med.

[17] Rosenberg ZS, Beltran J, Bencardino JT (2000). MR imaging of the ankle and foot. Radiographics.

[18] Wang X-T, Rosenberg ZS, Mechlin MB, Schweitzer ME (2005). Normal variants and diseases of the peroneal tendons and superior peroneal retinaculum: MR imaging features. Radiographics.

[19] Ma GMY, Ecklund K (2017). MR imaging of the pediatric foot and ankle: what does normal look like?. Magn Reson Imaging Clin N Am.

[20] Berman Z, Tafur M, Ahmed SS, Huang BK, Chang EY (2017). Ankle impingement syndromes: an imaging review. Br J Radiol.

[21] Szaro P, Polaczek M, Świątkowski J, Kocoń H (2020). How to increase the accuracy of the diagnosis of the accessory bone of the foot?. Radiol Med.

[22] Crim JR, Kjeldsberg KM (2004). Radiographic diagnosis of tarsal coalition. AJR Am J Roentgenol.

[23] Docquier P-L, Maldaque P, Bouchard M (2019). Tarsal coalition in paediatric patients. Orthop Traumatol Surg Res.

[24] Newman JS, Newberg AH (2000). Congenital tarsal coalition: multimodality evaluation with emphasis on CT and MR imaging. Radiographics.

[25] Lazzarini KM, Troiano RN, Smith RC (1997). Can running cause the appearance of marrow edema on MR images of the foot and ankle?. Radiology.

[26] Collins MS, Schaar MM, Wenger DE, Mandrekar JN (2005). T1-weighted MRI characteristics of pedal osteomyelitis. AJR Am J Roentgenol.

[27] Khanna G, Sato TSP, Ferguson P (2009). Imaging of chronic recurrent multifocal osteomyelitis. Radiographics.

[28] Fernandez-Canton G, Casado O, Capelastegui A, Astigarraga E, Larena JA, Merino A (2003). Bone marrow edema syndrome of the foot: one year follow-up with MR imaging. Skelet Radiol.

[29] Maillefert JF, Dardel P, Cherasse A, Mistrih R, Krause D, Tavernier C (2003). Magnetic resonance imaging in the assessment of synovial inflammation of the hindfoot in patients with rheumatoid arthritis and other polyarthritis. Eur J Radiol.

[30] Erdem CZ, Sarikaya S, Erdem LO, Ozdolap S, Gundogdu S (2005). MR imaging features of foot involvement in ankylosing spondylitis. Eur J Radiol.

[31] Mutlu H, Sildiroglu H, Pekkafali Z, Kizilkaya E, Cermik H (2006). MRI appearance of retrocalcaneal bursitis and rheumatoid nodule in a patient with rheumatoid arthritis. Clin Rheumatol.

[32] McQueen FM, Ostendorf B (2006). What is MRI bone oedema in rheumatoid arthritis and why does it matter?. Arthritis Res Ther.

[33] Erdem CZ, Tekin NS, Sarikaya S, Erdem LO, Gulec S (2008). MR imaging features of foot involvement in patients with psoriasis. Eur J Radiol.

[34] Kucera T, Shaikh HH, Sponer P (2016) Charcot neuropathic arthropathy of the foot: a literature review and single-center experience. J Diabetes Res 2016(3207043) 10.1155/2016/3207043 10.1155/2016/3207043 PMC5021483 27656656

[35] James SLJ, Panicek DM, Davies AM (2008). Bone marrow oedema associated with benign and malignant bone tumours. Eur J Radiol.

[36] Shabshin N, Schweitzer ME, Morrison WB, Carrino JA, Keller MS, Grissom LE (2006). High-signal T2 changes of the bone marrow of the foot and ankle in children: red marrow or traumatic changes?. Pediatr Radiol.

